# SonicParanoid2: fast, accurate, and comprehensive orthology inference with machine learning and language models

**DOI:** 10.1186/s13059-024-03298-4

**Published:** 2024-07-25

**Authors:** Salvatore Cosentino, Sira Sriswasdi, Wataru Iwasaki

**Affiliations:** 1 Department of Integrated Biosciences, Graduate School of Frontier Sciences, the University of Tokyo, Kashiwa, Japan; 2 Center of Excellence in Computational Molecular Biology, Faculty of Medicine, Chulalongkorn University, Bangkok, Thailand; 3 Department of Biological Sciences, Graduate School of Science, the University of Tokyo, Bunkyo-ku, Japan; 4 Department of Computational Biology and Medical Sciences, Graduate School of Frontier Sciences, the University of Tokyo, Kashiwa, Japan; 5 Atmosphere and Ocean Research Institute, the University of Tokyo, Kashiwa, Japan; 6 Institute for Quantitative Biosciences, the University of Tokyo, Bunkyo-ku, Japan; 7 Collaborative Research Institute for Innovative Microbiology, the University of Tokyo, Bunkyo-ku, Japan

**Keywords:** Orthology inference, Machine learning, Language model, Genome evolution

## Abstract

Accurate inference of orthologous genes constitutes a prerequisite for comparative and evolutionary genomics. SonicParanoid is one of the fastest tools for orthology inference; however, its scalability and accuracy have been hampered by time-consuming all-versus-all alignments and the existence of proteins with complex domain architectures. Here, we present a substantial update of SonicParanoid, where a gradient boosting predictor halves the execution time and a language model doubles the recall. Application to empirical large-scale and standardized benchmark datasets shows that SonicParanoid2 is much faster than comparable methods and also the most accurate. SonicParanoid2 is available at https://gitlab.com/salvo981/sonicparanoid2 and https://zenodo.org/doi/10.5281/zenodo.11371108.

**Supplementary Information:**

The online version contains supplementary material available at 10.1186/s13059-024-03298-4.

## Background

The accurate inference of orthologous genes originating from speciation events is crucial in various areas of genomics and evolutionary biology [1]. Many tools and resources have been developed to identify orthologous relationships among multiple proteomes. They are classified into graph [2–6] and tree based [7–9], which do not include tools that integrate both approaches (hybrid) or integrate publicly available resources to perform their predictions [10, 11]. Graph-based tools infer orthologs by calculating all-versus-all pairwise similarity scores and using the bidirectional-best-hit (BBH) method or its derivatives [12]. In tree-based methods, orthologs are identified by analyzing phylogenetic trees, which allows the identification of speciation and duplication events. Tree-based tools are more computationally demanding and are typically available to users only via databases or web services. A comparative study showed no significant differences in the ability of the two approaches in inferring orthologs [13].

The steep decrease in sequencing costs and the associated increase in genomic and metagenomic data [14] challenge the scalability of orthology inference tools. Inferring orthologs de novo for a few hundred proteomes using graph-based methods on high-performance computing servers may require days to weeks, even when local alignment tools, such as MMseqs2 [15] and Diamond [16], are used as faster alternatives to BLAST [17].

In addition to the scalability problem, graph-based methods tend to miss orthologs in duplication-rich proteomes [18] (e.g., plant proteomes) and in proteins with complex domain arrangements (architectures) originating from fusion and fission events [19, 20]. For example, BBH-based methods fail to identify human kinase orthologs detected using a domain-based orthology inference method [21]. The importance of orthology-inference at the domain level was also highlighted by its recent integration into the long-standing InParanoidDB [22].

Machine learning (ML), particularly methods borrowed from natural language processing (NLP), has been extensively used in genomics [23]. Currently, deep learning and language models are used in multiple areas of genomics [24], including sequence assembly and binning [25, 26] as well as protein folding [27, 28]. ML is gradually being adopted for orthology inference [29, 30]; however, the sensitivity, scalability, and usability of ML-based methods can be further improved.

Herein, we report a major update to SonicParanoid [31], which is one of the fastest de novo orthology inference tools [30, 32]. The update uses two ML methods, AdaBoost [33] and Doc2Vec [34], to deliver a substantially faster, more accurate, and more comprehensive orthology inference.

## Results

### SonicParanoid2 as the fastest and most accurate orthology inference method

**Fig. 1:**
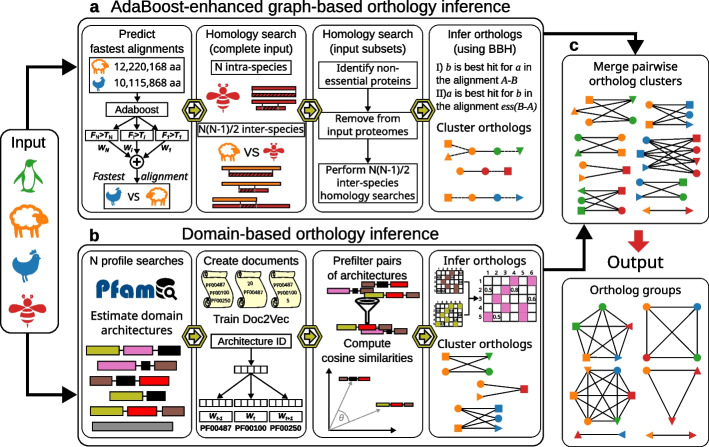
Overview of SonicParanoid2. **a** Graph-based orthology inference pipeline using a novel ML-based approach to substantially reduce execution time of homology searches. **b** Domain-based orthology inference pipeline that compares domain architectures using methods from NLP. **c** Pairwise orthologous tables predicted using the two pipelines combined to generate ortholog graphs from which the output ortholog groups are inferred

For a set of *N* proteomes, SonicParanoid2 performs de novo orthology inference using a novel graph-based algorithm that halves the execution time with an AdaBoost classifier and avoiding unnecessary alignments (Fig. 1a). Furthermore, for a more comprehensive identification of orthologs, SonicParanoid2 conducts domain-based orthology inference using Doc2Vec neural network models (Fig. 1b). The clusters of orthologous genes from each species pair (the species-species ortholog clusters) predicted by these two algorithms are merged (Fig. 1c) and input into the Markov cluster algorithm [35] (MCL) to infer the multi-species ortholog groups [2] (OGs) for the *N* input proteomes. SonicParanoid2 can be executed using three predefined modes (fast, default, and sensitive), in which different local alignment tools and settings are used (Additional file 1: Table S1).

The effect of domain-based orthology on the number of predicted orthologs and the total execution time differs depending on the input dataset and settings for SonicParanoid2. For example, on a dataset provided by the Quest for Orthologs [36, 37] (QfO) (Additional file 1: Fig. S1), the number of predicted orthologs increased by one third at the default settings, and the total execution time showed a similar increase (26.86%) (Table 1). The increase in the total execution time becomes relatively negligible for larger datasets and at higher sensitivity settings because of the high scalability of the domain-based pipeline (Additional file 1: Fig. S2). For example, on a dataset with 2000 metagenome-assembled genomes (MAGs) (Additional file 1: Fig. S3), the increases in execution time were 16% and 13% for the default and fast modes, respectively, whereas one third more orthologs were predicted regardless of the settings (Table 2). The execution time of the domain-based algorithm is almost the same regardless of the mode at which SonicParanoid2 is executed, and so its impact on the total execution time varies depending on the time the graph-based pipeline took. For example, on a dataset of 200 eukaryotes (Additional file 1: Fig. S4), the domain-based algorithm increases the execution time by 28% and 2% for the fast and sensitive modes, respectively (Table 3).

**Table 1:** Execution times of SonicParanoid2 and effects of including domain-based orthology. Results were obtained using the QfO dataset as input. Execution time columns show the total execution time and its increase due to the execution of the domain-based pipeline. Increase in predicted orthologs provided by the domain-aware pipeline is up to one third (columns of predicted ortholog pairs). Last four columns show the speedup folds relative to the execution times of the compared methods

	Execution time		Predicted ortholog pairs		Speed-up folds on other methods			
Mode	Total (hours)	Increase(%)	Count (million)	Increase (%)	Broccoli	OrthoFinder	OrthoFinder (MSA)	ProteinOrtho
Default	0.83	26.86	15.28	27.39	3.57	1.64	11.91	− 1.19
Fast	0.55	49.99	14.64	29.79	5.41	2.49	18.08	1.27
Sensitive	4.28	4.29	19.27	23.14	− 1.44	− 3.13	2.32	− 6.12

**Table 2:** Effects of including domain-oriented orthology when processing a set of 2000 MAGs. Execution time columns show the total execution time and its increase due to the inclusion of the domain-based pipeline. The increase in execution time is low if we consider the magnitude of the input dataset, whereas the number of predicted orthologs increased by more than one third. OrthoFinder and Broccoli failed to complete the execution for different reasons

	Execution time		Predicted ortholog pairs		Speed-up folds
Mode	Total (hours)	Increase (%)	Count (million)	Increase (%)	ProteinOrtho
Default	41.60	15.69	1817.40	30.66	3.29
Fast	25.03	13.56	1694.88	37.39	5.47

**Table 3:** Effects of including domain-oriented orthology when processing a set of 200 eukaryotic proteomes. Execution time columns show the total execution time and its increase due to the inclusion of the domain-based pipeline. Broccoli was using the whole 2 terabytes of memory in the system and was terminated

	Execution time		Predicted ortholog pairs		Speed-up folds on other methods			
Mode	Total (hours)	Increase (%)	Count (million)	Increase (%)	Broccoli	OrthoFinder	OrthoFinder (MSA)	ProteinOrtho
Default	5.74	13.58	148.06	21.67	NA	1.65	6.92	1.13
Fast	3.07	28.43	141.81	23.42	NA	3.09	12.93	2.11
Sensitive	38.85	1.67	181.25	17.15	NA	− 4.09	1.02	− 5.99

We compared the execution time of SonicParanoid2 with those of OrthoFinder [10], ProteinOrtho [38], and Broccoli [30], which are among the fastest tools for de novo orthology inference. Broccoli reduces the burden of all-versus-all alignments through *k*-mer clustering, while ProteinOrtho uses a heuristic approach to halve the number of required alignments. Multiple studies demonstrated that these tools are similarly accurate for inferring OGs [32, 39, 40].

On the QfO dataset, the fast mode of SonicParanoid2 was 5x, 2.5x, and 1.3x faster than OrthoFinder, Broccoli, and PorteinOrtho, respectively (Table 1). At the default settings, SonicParanoid2 was 3.6x and 1.6x faster than Broccoli and OrthoFinder, respectively, but slightly slower (− 1.2x) than ProteinOrtho. In the sensitive mode, SonicParanoid2 was slower than others due to the use of MMseqs2 at the highest sensitivity (Additional file 1: Table S1), but it was 2.3x faster than OrthoFinder (MSA), which performs additional multiple sequence alignments to increase the sensitivity. Furthermore, when the same sensitivity settings for Diamond are used for both ProteinOrtho and SonicParanoid2 a similar execution time was observed (Additional file 1: Table S2).

We also estimated the peak memory usage of each tool on the QfO dataset (Additional file 1: Table S3 and Supplementary text) and found ProteinOrtho to have the lowest memory footprint, while Broccoli has the highest. ProteinOrtho also exhibited very low CPU times since it performs only half of the alignments compared to SonicParanoid2 and OrthoFinder.

Orthologous relationships for the MAG dataset were inferred by SonicParanoid2 in 1.7 and 1 day in the default and fast modes, respectively (Table 2). Conversely, OrthoFinder and Broccoli failed to complete orthology inference on the MAG dataset for different reasons (Additional file 1: Supplementary text), while ProteinOrtho took 5.7 days (5.5x slower than SonicParanoid2).

**Fig. 2:**
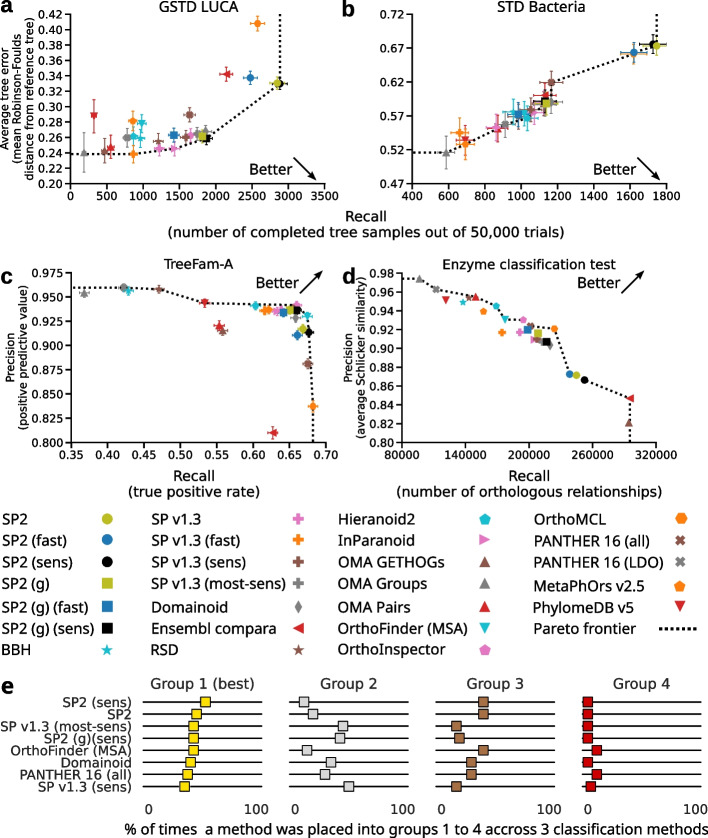
Accuracy of SonicParanoid2 compared with other 14 methods. **a**–**d** Accuracies of SonicParanoid2 (SP2) using different settings on four among 12 QfO 2020 benchmark tests. Methods lying on Pareto frontier indicate the best balance between precision and recall. Results obtained using only the novel graph-based approach (SP2 (g)) are shown (squares) for comparison with older versions of SonicParanoid (crosses). **a** Generalized species tree discordance on LUCA datasets. **b** Species tree discordance test on the Bacteria dataset. **c** Reference gene tree test based on TreeFam-A. **d** Functional benchmark test based on Enzyme Commission (EC) numbers. **e** Top eight methods according to the aggregate ranking from the three classification methods in the QfO benchmark

The accuracy of SonicParanoid2 was evaluated using the QfO benchmark [37] and compared with 14 well-established methods, including a legacy version of SonicParanoid [31]. In 12 tests performed, methods close to or constituting the Pareto frontier were regarded as those that provided the best tradeoff between precision and recall. SonicParanoid2 was Pareto optimal in multiple tests, including the LUCA and bacterial species tree discordance tests (Fig. 2a–d and Additional file 1: Figs. S5 and S6). Lastly, SonicParanoid2 is the most accurate method based on the aggregate ranking of three classification methods provided by the QfO benchmarking service (Fig. 2e and Additional file 1: Figs. S7-S10).

### Substantially faster and scalable graph-based orthology inference using machine learning

Graph-based de novo orthology inference for *N* proteomes generally requires *N* intra-proteome and *N(N-1)* bidirectional inter-proteome all-versus-all alignments. For the bidirectional inter-proteome alignment of proteomes A and B, all proteins in A are queried against proteome B (denoted as alignment A-B), and all proteins in B are queried against proteome A (B-A). The key rationale behind the way in which SonicParanoid2 reduces the computational cost is as follows: (1) the execution times of alignments A-B and B-A can substantially differ depending on the proteome size and evolutionary distance between A and B (Additional file 1: Figs. S11 and S12), and (2) query and target proteins that have no hits with bitscores above a predefined threshold in one of the two inter-proteome alignments cannot be predicted as orthologs, based on the definition of BBH (Eq. 1). Accordingly, for a pair of proteomes A and B, SonicParanoid2 first predicts the faster alignment between A-B and B-A using an adaptive gradient boosting [33] (AdaBoost) binary classifier (Additional file 1: Table S4). If A-B is predicted to be faster, then SonicParanoid2 first conducts the alignment A-B and creates two subsets, ess(A) and ess(B), which include only proteins with alignment scores above the threshold (Eq. 2) and representing the best-hits for the A-B alignment. Subsequently, the alignment between ess(B) and ess(A) is performed instead of the slower alignment of B-A (Additional file 1: Fig. S13). Finally, the algorithm identifies the orthologs as shown in Eq. 3. Henceforth, we will refer to the subsets of proteins generated by this graph-based algorithm as “essential subsets” and to the all-versus-all among these subsets as “essential alignments.”

**Fig. 3:**
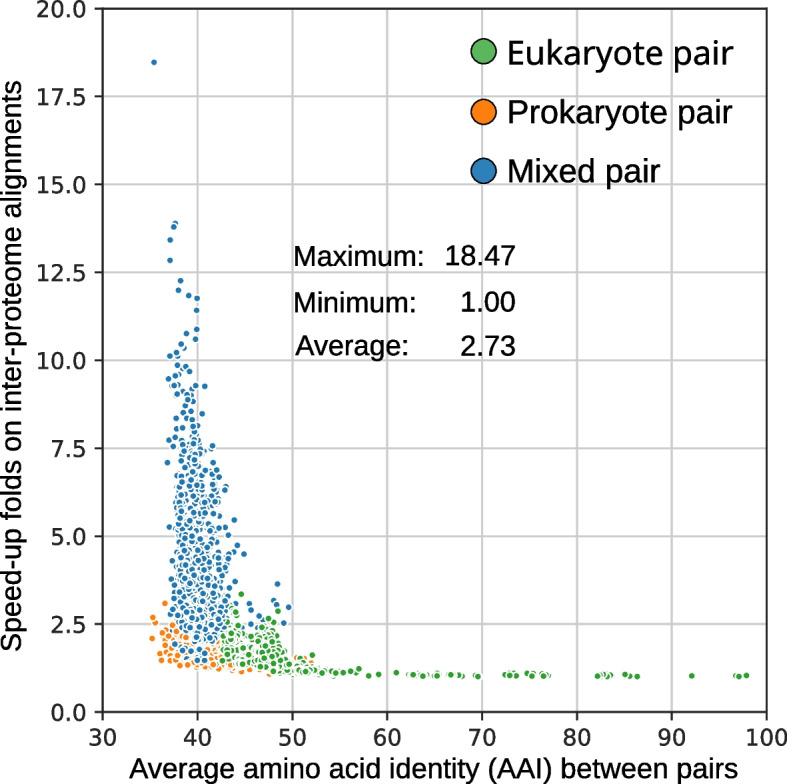
Speedup from essential alignments and evolutionary relatedness. For two proteomes A and B, the total execution time is computed as the sum of the execution times for the inter-proteome alignments A-B and B-A. The speedup (on the *Y*-axis) obtained for each bidirectional alignment was computed as the difference in total execution time required with and without using the essential alignments. The speedups obtained using the novel approach in SonicParanoid2 are inversely proportional to the evolutionary relatedness (expressed in terms of AAI) of the proteome pairs. The results above were obtained using the QfO dataset as input and MMseqs2 at the most sensitive settings

We evaluated the execution time of the novel graph-based algorithm in SonicParanoid2 (Fig. 1a) on the QfO and MAGs datasets (Additional file 1: Figs. S1 and S2) using different alignment tools and sensitivity settings (Additional file 1: Table S5). For the QfO dataset, the execution time for the all-versus-all alignments was reduced by 42% (Additional file 1: Table S6). Moreover, when only inter-proteome alignments were considered, the reduction in execution time was as high as 95% (Fig. 3). A speedup was observed regardless of the alignment tool used (MMseqs2, Diamond, or BLAST). Nevertheless, the extent depended on both the alignment tool used and its sensitivity settings (Additional file 1: Table S6). Specifically, the proportions of sequences used in the second alignment were smaller for the proteome pairs of phylogenetically distant species (Additional file 1: Fig. S14), thus resulting in higher speedups. For example, 97% of the input proteins was not used in the second alignment between *Leptospira interrogans* (bacteria) and *Giardia intestinalis* (protist), which consequently reduced the execution time substantially. Conversely, less than 2% of the original input was removed for the second alignment between closely related chimpanzee and gorilla proteomes.

The accuracy of the AdaBoost classifier differed depending on the alignment tool for which it inferred faster alignments, partly because it was trained for MMseqs2 (Additional file 1: Table S6). The necessity of the AdaBoost model was assessed by measuring the reduction in execution time when the slowest alignments were conducted first. This task was performed by inverting the predictions of the AdaBoost classifier. For example, when the classifier predicted A-B as the fastest alignment, we first aligned B-A. We observed that the amount of saved time was up to 70% less, which proved the importance of predicting the fastest inter-proteome alignments (Additional file 1: Table S7). Additionally, compared to randomly selecting the alignments to perform, following AdaBoost prediction was 7-22% faster depending on the sensitivity settings (Additional file 1: Table S8).

To evaluate the scalability of the graph-based algorithm of SonicParanoid2, we performed orthology inference on a large dataset comprising 2000 MAGs from aquatic and terrestrial environments (Additional file 1: Fig. S3). The required four million all-versus-all alignments were performed in 56.48 and 23.74 h using MMseqs2 and Diamond, respectively, which corresponded to execution times reduced by 16.18% and 24.99%, respectively (Additional file 1: Table S9). The MAG dataset contained many closely related microbial genomes, and the closely related proteomes therein limited the reduction in execution time (Additional file 1: Figs. S14 and S15).

### Speed and accuracy of the AdaBoost-enhanced graph-based method

We compared the execution times of the AdaBoost-enhanced graph-based algorithm (Fig. 1a) with those of Broccoli and OrthoFinder. In particular, the domain-based algorithm in SonicParanoid2 was disabled. On the 78-proteome QfO dataset, the fast mode of SonicParanoid2 using Diamond (Additional file 1: Table S5) completed the execution in 0.38 h, whereas OrthoFinder and Broccoli took 10.19 and 2.77 h (7.3x and 26.9x slower), respectively (Additional file 1: Table S10). Subsequently, we tested OrthoFinder and Broccoli on the 2000-proteome MAG dataset; however, both tools failed to complete the orthology inference for different reasons (Additional file 1: Supplementary text), whereas SonicParanoid2 required one day (Additional file 1: Table S9).

To compare the AdaBoost-enhanced method with the conventional graph-based approach, which uses all the input sequences, we evaluated the prediction sets of 18 trials (Additional file 1: Table S6) using the QfO benchmark. The accuracies of the two graph-based approaches were highly similar, with no apparent adverse effect on the accuracy despite the substantial reduction in computational time (Additional file 1: Figs. S16 and S17). This can better be seen in Additional file 1: Figs. S7-S10 in which “SP v1.3 (most-sens)” and “SP2 (g) (sens)” are ranked next to each other in three of the four rankings. These two methods used only the graph-based algorithm and the same settings for MMseqs (Additional file 1: Tables S1 and S5).

### Fast and scalable domain-based orthology inference using Doc2Vec

**Fig. 4:**
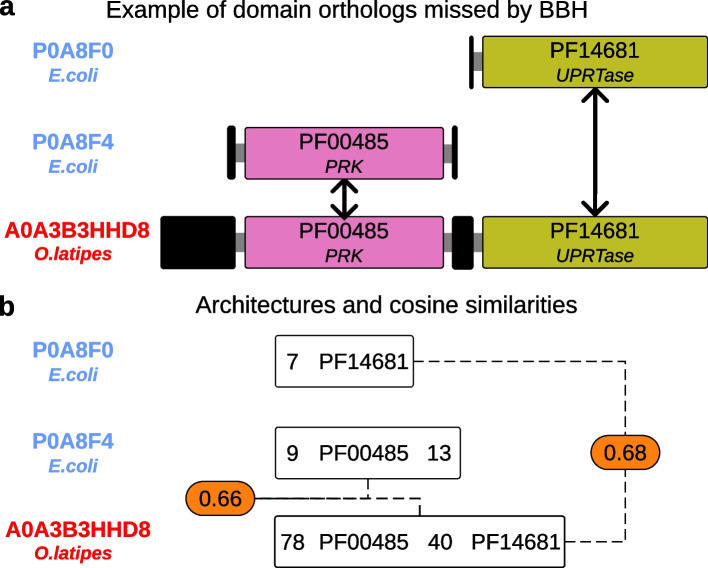
Recovery of orthologs missed by BBH via domain-based orthology inference. **a** Protein A0A3B3HHD8 from *O. latipes* is a representative example of domain fusion resulting from two single-domain *E. coli* proteins. Pink and green boxes in **a** are functional domains (annotated with their Pfam ID and gene name), whereas black boxes represent inter-regions with no Pfam annotation in InterPro (as of January 2023). P0A8F4 and P0A8F0 are orthologous to A0A3B3HHD8 (black arrows), but the graph-based pipeline identifies only P0A8F4 as an ortholog. **b** Using domain-aware orthology, SonicParanoid2 predicts P0A8F0 as an ortholog of A0A3B3HHD8. Text inside the white boxes represent the architectures for the proteins above as they appear in the training corpus for the Doc2Vec model. The rounded rectangles in orange show the cosine similarities assigned to the two pairs of domain architectures

The graph-based approach based on BBH can miss orthologs of proteins with many duplications or those that have undergone domain fusion or fission [18, 20, 21] (Fig. 4a and Additional file 1: Fig. S18). Thus, in addition to the orthologs predicted using the graph-based approach, SonicParanoid2 infers orthologs at the domain level by comparing functional domain architectures using techniques typically used in natural language processing (Fig. 1b and Fig. 4b).

SonicParanoid2 uses fast profile searches on Pfam [41] to infer the domain architectures of the input proteins and converts them into “phrases,” where “words” are the annotated functional domains and the amino-acid lengths of the inter-domain regions (Fig. 1b). The phrases are used as the training corpus for a Doc2Vec [34] neural network model that associates a numerical vector with each domain architecture. For each inter-proteome pair, after a filtering step, the cosine similarities of the inter-proteome domain architecture pairs are computed. Pairs of architectures with cosine similarities above a predefined threshold are considered candidate orthologs.

The increase in total execution time due to the inclusion of the domain-based pipeline was negligible, considering the increase in the number of predicted orthologs (Tables 1, 2, and 3). Due to the high scalability of the domain-based pipeline (Additional file 1: Fig. S2), the aforementioned smallness of the increase in execution time was more apparent when processing larger datasets. For example, for the MAG dataset, the increases in execution time were 16% and 13% for the default and fast modes, respectively, whereas the increase in the number of predicted orthologs was about one third, regardless of the settings (Table 2). The impact on the total execution time is even lower when SonicParanoid2 is executed in the sensitive mode (Tables 1 and 3, only 1.7–4.3% increases). The scalability of the method also allows us to train the Doc2Vec model on the fly with documents (architectures) extracted from the input dataset. This ensures that the model has seen the entire corpus and can provide an embedding vector for each domain architecture, thus enabling faster executions.

The inclusion of the domain-based pipeline substantially increased the recall. For example, in the bacterial species tree discordance test, the recall doubled in some settings (Additional file 1: Figs. S19 and S20). Nevertheless, the additional orthologs obtained from the domain-based pipeline might contain false positives. This may explain the result on the SwissTree dataset (Additional file 1: Fig. S6a, the right panel) in which SonicParanoid2 obtained a small increase in recall at the cost of precision. It should be noted that at present the QfO benchmark service does not fully support predictions obtained at the domain level, although the 2022 challenge (for which results are publicly available) includes a new test that scores domain architectures of the predicted orthologs using the approach described in Dosch et al. (2023) [42]. In this test, the inclusion of the predictions obtained using Doc2Vec resulted in an increase in both precision and recall.

The embeddings generated by Doc2Vec are sensitive to the order of the elements in the architectures (annotated domains and unannotated regions), but this comes with limitations. For example, the embeddings for architectures that have experienced domain shuffling or other rearrangement would have low cosine similarities and would ultimately be rejected by our algorithm. Domain rearrangement events could potentially be detected by replacing Doc2Vec with attention-based methods [43], or by including metrics that are order-invariant, such as the Jaccard index in Domainoid [20], or by allowing each domain to have different orthology. We acknowledged the limitation of our approach and used a very conservative approach when including predictions from the domain-based algorithm. This partially explains why the biggest improvement in recall when the domain-based algorithm was used was observed in the STD Bacteria dataset, which likely contain simpler architectures compared to other test datasets (Additional file 1: Fig. S2b).

The overall accuracy was significantly increased, making SonicParanoid2 the most accurate based on three classification methods provided by the QfO benchmark service (Fig. 2e and Additional file 1: Figs. S7-S10). More importantly, such boost in accuracy is higher when SonicParanoid2 is executed at lower sensitivities, allowing for accurate predictions with shorter runtimes (Additional file 1: Table S11).

The recovery of eukaryotic uridine–cytidine kinases are examples of orthologous relationships recovered using domain-based orthology inference. Previous studies showed that the fusion of prokaryotic phosphoribulokinase/uridine kinase and uracil phosphoribosyltransferase domains (P0A8F4 and P0A8F0) resulted in the emergence of this enzyme in human [20] (UniProt ID Q9NWZ5). Whereas the graph-based method of SonicParanoid2 identified P0A8F4 only as an ortholog of Q9NWZ5, the domain-based method identified P0A8F0 as well. Additionally, the domain-based method predicted P0A8F0 to be an ortholog of other 17 eukaryotic uridine–cytidine kinases in 12 eukaryotic species in the QfO dataset (Additional file 1: Table S12).

## Conclusion

Fast, accurate, and comprehensive identification of orthologous genes is becoming increasingly important in genomic and evolutionary studies owing to the steady growth of publicly available genomic data [14]. Nevertheless, existing de novo orthology inference tools require long execution times because of the necessary all-versus-all alignments and cannot effectively detect orthologs of proteins with complex domain architectures or those that have undergone gene fusion or fission events [20, 21].

SonicParanoid2 solves the two aforementioned problems using ML and is faster and more accurate than existing tools. The novel graph-based algorithm almost halves the execution time by avoiding unnecessary homology searches and does not degrade the accuracy. The domain-aware algorithm increased the number of predicted orthologs by one third and significantly increased the accuracy with minimal costs for the total execution time. Evaluation based on standardized benchmarks showed that SonicParanoid2 is the most accurate among other 14 well-established methods.

While SonicParanoid2 was the fastest thanks to several optimizations in the graph-based pipeline, it could be further improved. The AdaBoost model for selecting the faster inter-proteome alignment to perform could be substituted with a simple heuristic approach based on proteome size. Additionally, the “pseudo-reciprocal best alignment” approach used by ProteinOrtho has a lot of potential, if it could be improved to lower the negative impact on accuracy and integrated with our techniques.

Considering the way SonicParanoid2 uses language models to infer orthologs, and their recent applications in bioinformatics (e.g., ProtVec [44], SeqVec [45] and ESMFold [28]), we expect accurate alignment-free orthology inference tools to be realized in the near future, which will result in significant advancements in comparative and evolutionary biology. Attention-based models have the potential to be able to identify orthologs that experienced complex domain shuffling events and to better incorporate domain-level orthology information into the embeddings.

## Material and methods

### Test datasets, accuracy benchmark, and ranking

The execution times of SonicParanoid2, Broccoli, and OrthoFinder were evaluated using the 2020 version of a benchmark dataset provided by the QfO consortium [37]. This dataset contains 78 proteomes (Additional file 1: Fig. S1a). Because it includes both closely and distantly related species, it provides a good example on the manner by which evolutionary relatedness affects the AdaBoost-enhanced graph-based algorithm in SonicParanoid2 (Additional file 1: Fig. S14).

The scalability of SonicParanoid2 was evaluated on a dataset comprising 2000 microbial MAGs from terrestrial and aquatic environments (Additional file 1: Fig. S3), which were randomly selected from a public catalogue of 52,515 MAGs [46]. The MAGs in the dataset satisfied or exceeded the medium quality level of the minimum information about metagenome-assembled genome (MIMAG) standard [47] and contained 5.1 million proteins. The final dataset contained 1786 pairs of MAGs (involving 931 MAGs) with an average amino acid similarity (AAI) exceeding 99%, suggesting that for approximately a quarter of the MAGs there is another MAG which is almost identical. Although we processed these MAGs, we recommend removing highly similar proteomes from the input dataset to avoid bias in the orthology inference. Additionally, we tested all methods on 200 eukaryotes reference proteomes from UniProt (Additional file 1: Fig. S4 and Additional file 2: Table S13) which is composed of 2.9 million proteins. This dataset is a subset of the one described in Additional file 1: Fig. S21.

To evaluate the accuracy of SonicParanoid2, the orthologs predicted for the QfO dataset were uploaded to a benchmark service provided by the QfO. The benchmark provides 12 tests [37]: two are based on reference gene trees, two use functional information, one is based on manually curated sets of orthologous relationships among mammals, and seven assess the validity of predictions in terms of the accuracy of the species trees that can be reconstructed from them. In most of the tests, small subsets of the uploaded predictions were used, except for the LUCA generalized species tree discordance test, which evaluated all predictions.

The accuracy of SonicParanoid2 was compared with those of other 14 methods, the results of which are publicly available on the benchmark web page (as of February 2023). Benchmark results for Broccoli are not available; furthermore, because only the developers of each tool can render the benchmark results publicly available on OpenEbench [48], the accuracy of Broccoli is not discussed herein. For ProteinOrtho6, the benchmark results were not available on the public server at the time that we prepared the materials. Currently, its performances at various settings can be found in Klemm et al. [37] and on the benchmark web page.

The ranking of the 15 methods into 4 groups (Fig. 2e and Additional file 1: Figs. S7-S10) was done based on the three classification methods (diagonal quartiles, K-means clustering, square quartiles) offered by the QfO benchmark services. Specifically, the diagonal quartiles approach ranks the methods by the distance from the “optimal performance” corner (denoted by an arrow in the plot). The K-mean clustering approach clusters methods with similar performances into 4 groups and sorts them. The square quartiles approach ranks the methods based on their relative performances compared to the median along the two metrics on the *X*-axis and *Y*-axis, with preference given to the precision over recall. Details on how to reproduce the ranking plots and the source scripts for the three classification methods can be found in Additional file 1: Supplementary text. Each participant was ranked counting how many times it was placed into groups 1 to 4, where group 1 contained methods with the highest performance. The rankings shown in Fig. 2e and Additional file 1: Fig. S7 were obtained by aggregating the ranks from the 3 classification methods. It should be noted that because the square quartiles classification preferentially rewards methods with higher precision, its rankings are significantly different from the other two classification methods.

### Software, databases, and settings

To perform a comparison of the execution times on the QfO 2020 and the MAG datasets, we used SonicParanoid 2.0.4 (commit bf30cb27), Broccoli 1.2 (commit 032064c), and OrthoFinder 2.5.4 (commit 1b3f37c). For the results regarding only graph-based orthology inference, SonicParanoid2 was executed with the option “--graph-only” to omit domain-based predictions and sensitivity settings, as shown in Additional file 1: Table S1. The additional parameters (if any) used for each tool and each run described in this study can be found in Additional file 1: Table S3. To evaluate the AdaBoost-enhanced graph-based method, SonciParanoid2 was executed using the settings described in Additional file 1: Table S5, which were used in older versions of SonicParanoid (up to v1.3.8).

OrthoFinder and Broccoli were implemented in the Python3 programming language, whereas SonicParanoid2 was implemented in Python3 and Cython [49] (v3.0.0a10). ProteinOrtho was implemented in the Perl, C++, and Python languages. All tests were performed using Python 3.8.10, while ProteinOrtho also required Perl (v5.38.2 was used). The execution times (wall times) and CPU times were measured using Hyperfine (https://github.com/sharkdp/hyperfine), while the peak memory usage were measured using the memory-profiler software tool (https://pypi.org/project/memory-profiler). More details can be found in Additional file 1: Supplementary text.

The alignment tools used in this study were MMseqs2 [15] (13-45111), Diamond [16] 2.0.12, and BLAST [17] 2.12.0. Additional file 1: Table S5 shows the mapping of the sensitivity of the alignment tools to the sensitivity settings of the tested orthology inference tools.

The average AAIs of proteome pairs in the QfO and MAGs datasets were computed using CompareM 0.1.2 (https://github.com/dparks1134/CompareM).

The profile database for Pfam [41] (version 35) was retrieved using the command *databases* in MMseqs2 with “Pfam-A.seed*”* as a parameter and indexed using the *createindex* command with parameters “-k = 5” and “-s = 7.”

The Doc2Vec model was trained using the Gensim [50] (ver. 4.2) library for Python3.

### Graph-based orthology inference

The graph-based orthology inference in SonicParanoid2 uses AdaBoost and exploits the properties of the original BBH (Eq. 1) to reduce the execution time required by all-vs-all alignments.

1 $$\{\begin{array}{c} c{\text{I}})\,{b}\ {\text{is best hit for query}}\ {a}\ {\text{in the alignment}}\ A{\text{-}}B\\ {\text{II}})\,{a}\ {\text{is best hit for query}}\ {b}\ {\text{in the alignment}}\ B{\text{-}}A\end{array}$$

The workflow of graph-based orthology inference (Fig. 1a) for *N* input proteomes is shown in Additional file 1: Fig. S13. The first step involves predicting the *N(N-1)/2* fastest inter-proteome alignments using the AdaBoost binary classifier. Next, local alignments of the proteome pairs predicted to have the shortest execution time are performed using all input proteomes. Only the results for the best hits were kept. For each of these alignments, let us assume A-B, the best hits are processed to create two essential subsets (*ess(A)* and *ess(B)*) of the original proteomes using the conditions shown in Eq. 2.

2 $$\{\begin{array}{c}c{\text{I}}^{\prime})\,\forall\,{a}\in\ {A}\ \exists\;{\text{at least one target}}\ {b}\in\ {B}:\ {score}(a, b) \geq\ {t}\\ {\text{II}}^{\prime})\,\forall\,{b}\ \in\ {B}\ \exists\;{\text{at least one target}}\ {a}\ \in\ {A}:\ {score}(b,a)\geq {t}\end{array}$$

These subsets are used for the remaining *N(N-1)/2* inter-proteome alignments. *N* intra-proteome alignments are performed in parallel using complete proteome sets.

Once all local alignments are completed, their outputs are processed, and the graph-based ortholog relations are inferred using Eq. 3 and clustered as in the original SonicParanoid [30] algorithm (Additional file 1: Supplementary text).

3 $$\{\begin{array}{l}c{\text{I}})\,{b}\,{\text{is best hit for query}}\,{a}\ {\text{in the alignment}}\,A{\text{-}}B\\ {\text{II}})\,{a}\ {\text{is best hit for query}}\,{b}\,{\text{in the alignment}}\ {ess}(B){\text{-}}ess(A)\end{array}$$

### AdaBoost training and optimization

The AdaBoost [33] binary classifier was built using 62,250 training samples containing properties extracted from 250 reference proteomes (Additional file 1: Fig. S21 and Additional file 3: Table S14) and labeled using the execution times of inter-proteome alignments performed using MMseqs2 at the highest sensitivity. Given *N* input proteomes, for each of the *N(N-1)* inter-proteome alignments, we created a training sample with properties such as protein count, proteome size, and average protein length. Additionally, we included the differences in proteome size and protein count, expressed in folds, by assuming that the query proteome (leftmost in the pair) was smaller and had fewer proteins than the target proteome. Each sample was labeled based on the execution times of the two inter-proteome alignments of the proteome pair (Additional file 1: Table S4). As an example, for a sample representing A-B, if the execution time for its alignment is shorter than that of the other alignment (B-A), then its label is set to 1 (faster); otherwise, it is set to 0 (slower).

We selected the features in Additional file 1: Table S4 because, for two proteomes (A and B) with substantially different compositions, the execution times for the inter-proteome alignments can differ significantly. More specifically, if proteome A contains much fewer sequences (or amino acid bases) than proteome B, then the time required to align A-B may be much shorter than that to align B-A. Furthermore, because the difference in execution time was directly proportional to the difference in the composition of the proteomes, regardless of the alignment tool used (Additional file 1: Figs. S11 and S12); this information was used as the training feature of the AdaBoost classifier. Finally, because this information is computed and used at each execution by SonicParanoid2, regardless of the binary classifier, the creation of samples to predict the fastest alignments has virtually no overhead.

Based on the accuracies of the model on Diamond and MMseqs2 (Additional file 1: Table S6) as well as considering the estimated execution time for labeling the training samples using BLAST (which is likely more than 2 months), we decided to use only the model trained on samples labeled using MMseqs2.

The training, validation, and optimization of the model were conducted using AdaBoost libraries in Scikit-learn [51]. Hyperparameter optimization was performed using a grid search, where the best performing model achieved a mean test accuracy of 97.90 in a 10-fold cross validation. For the independent QfO test dataset, the maximum accuracy achieved by the model was 96.70% (Additional file 1: Table S6). Based on observed association between proteome sizes and execution times (Additional file 1: Fig. S12), we also investigated the possibility of using the proteome size as a heuristic for selecting the faster inter-proteome alignment to be performed. Specifically, by setting the larger proteome to be the query and the smaller proteome to be the target, the execution times of the alignments were comparable (only 1–3% difference) to those obtained when using the AdaBoost model. Although we tested this only on the QfO dataset, it might be a viable substitute for the AdaBoost model.

### Construction of domain architectures and training *corpus* for Doc2Vec

Examples of architecture estimation and document creation for a single protein are depicted in Additional file 1: Fig. S22. Hereinafter, we will use the terms “document” and “architecture” interchangeably and similarly for “word” and “annotated/unannotated domains.” The first step in estimating the architectures involves searching for input proteins in a Pfam profile database using MMseqs2. The hits are filtered to obtain a bit score and target coverage of at least 30 and 75%, respectively. For queries with multiple domains, the architectures are composed of non-overlapping domains and inter-regions for which no annotation was identified (uncovered regions). The elements of the architectures are converted into words and the architectures into phrases (documents). In the documents, annotated domains are represented by their Pfam annotations and uncovered regions (longer than four amino acid) by their lengths (Additional file 1: Fig. S22). A document constitutes the training corpus if it has a protein coverage (the proportion of the protein sequence annotated with domains) of at least 70% and is not repeated in the corpus.

### Doc2Vec model training

Doc2Vec (also known as Paragraph2Vec) is an unsupervised learning method that is an extension of Word2Vec [52] which represents a document as a numerical vector [34]. It uses Word2Vec to generate embeddings of single words; therefore, the embeddings can be learned through a continuous bag-of-words or skip-gram algorithm. In Doc2Vec, an algorithm that is similar to skip-gram is known as the “distributed bag-of-words of the paragraph vector” and is used to train the Doc2Vec models in SonicParanoid2. The context window value for the skip-gram was set to two, the number of dimensions (vector size) was set to 100, and training was performed for 200 epochs. Although these parameter settings are typically used in studies pertaining to NLP [53], we set the minimum word frequency to one. This parameter controls the minimum number of times a certain word must appear in the corpus in order to be used for training. Because NLP models are typically trained on billions of documents [23], this parameter is often set to values equal to or higher than five to reduce the training time and memory usage. Nevertheless, in our case, the training sets were relatively small, where 134,520, 285,092, and 567,119 documents were obtained for the QfO, the 200 eukaryotes, and MAG datasets, respectively. Hence, we set the minimum word frequency to one, allowing the neural networks of the Doc2Vec model to be trained using all documents in the corpus. The training is performed on a single CPU core for reproducibility and typically finishes in minutes (Additional file 1: Fig. S2). It should be noted that every time SonicParanoid2 is run, the Doc2Vec model is trained on the fly on the corpus generated from the input proteomes and the trained model is used only on those proteomes. This also ensures that the model recognizes every annotated domain architecture in the input dataset.

### Architecture prefiltering and creation of domain-based ortholog clusters

Given proteomes A and B with *l* and *m* architectures, respectively, *l* x *m* pairs of architectures exist. Accordingly, computing the cosine similarities for all the possible pairs of architectures for the *N(N-1)/2* combinations of input proteomes will be extremely computationally demanding. To reduce the execution time, SonicParanoid2 performs prefiltering, in which pairs of architectures that are unlikely to be orthologous are rejected before their cosine similarities are computed.

Filtering is performed by comparing features of the architectures, including the protein length, protein coverage, and number of annotated domains (Additional file 1: Fig. S22c).

A pair of architectures is rejected if one of the following conditions applies:

1. The protein length of one architecture exceeds 3X the length of the other
2. Their protein coverages differ by more than 25%
3. One of the two architectures has more than double the domains of the other
4. The two architectures have no mutual domains

After prefiltering, the cosine similarities between the remaining pairs of architectures are computed, and cosine similarity values of at least 0.5 populate a matrix M of dimension *l* x *m*. The domain-based algorithm generates clusters of orthologs by selecting the maximum cosine similarities in M for architectures from A (row-wise) and B (column-wise) proteomes (Additional file 1: Fig. S23).

### Merging of pairwise orthologs and inference of OGs

The pipelines depicted in Fig. 1a and b infer orthologous relationships between pairs of species using the graph- and domain-based algorithms, respectively. SonicParanoid2 combines these orthologs to generate ortholog graphs, which are subsequently used to infer OGs (Fig. 1c). Because we regarded graph-based predictions as more accurate, we integrated domain-based predictions into them.

Let G and D be sets of species-species ortholog clusters for proteomes *A* and *B* generated by the graph- and domain-based algorithms, respectively. Let g ∈ G and d ∈ D be clusters of ortholog relationships. When merging the two sets of predicted orthologs, we considered the following three cases:

1. *d* is a completely new cluster with no proteins contained in any cluster in *G*
2. All proteins in d are already predicted as orthologs in other clusters in *G*
3. Some proteins in *d* are not in any cluster in *G*, and some proteins in g are not in any *d ∈ D*

In case 1, the architecture of each ortholog in *d* must have a protein coverage of at least 75%; otherwise, it is rejected. Additionally, if even a single pair of architectures has a different number of annotated domains, cluster *d* is rejected completely; cluster *d* is added as a new cluster in *G* if none of the aforementioned applies.

In case 2, we prioritized graph-based predictions and use the clusters in *G*, which already contain the orthologs in *d*, without any modifications.

Case 3 is the most complicated and involves scenarios in which some proteins in d are contained in one or more clusters in *G*, whereas others are not. In this case, the orthologous proteins from *d* with coverages higher than or equal to 75% are integrated into the existing *G* clusters. One such example is the protein P0A8F4 (Fig. 4b). For proteins A0A3B3HHD8, P0A8F4, and P0A8F0, the graph-based cluster *g* ∈ *G* contains all proteins except P0A8F0. Cluster *d* contains all three proteins; therefore, P0A8F0 (with 96.2% coverage) is inserted as a new ortholog into the corresponding cluster in *G*.

Combined sets of pairwise orthologs are used to construct ortholog graphs, which are the input to the MCL. The output from the MCL is analyzed to extract the OGs, which is the final output of the SonicParanoid2 (Fig. 1c).

### Hardware used

The results described herein were obtained using an Ubuntu 20.04.04 LTS (Linux 5.15.0) HPC server, which was equipped with a 128 cores AMD EPYC 7742 CPU, 2 terabytes of memory, and a 3.5 terabytes solid state disk.

### Supplementary Information

Additional file 1: Supplementary text, figures (Figs. S1-S23), and tables (Tables S1-S12).

Additional file 2: Table S13: List of UniProt reference proteomes for the 200 eukaryotes test dataset.

Additional file 3: Table S14: List of UniProt reference proteomes for the 250 eukaryotes-prokaryotes dataset used to train the AdaBoost model.

Additional file 4. Peer review history.

## Data Availability

SonicParanoid2 is freely available with a GNU GPLv3 license in the Python Package Index (https://pypi.org/project/sonicparanoid) and GitLab (https://gitlab.com/salvo981/sonicparanoid2). An archive with the source code of the version of SonicParanoid used in this study (2.0.4) is available on Zenodo [54]. The documentation is available at https://gitlab.com/salvo981/sonicparanoid2/-/wikis/home. The benchmark results for SonicParanoid2 are publicly available at https://orthology.benchmarkservice.org/proxy/results/2020. The proteome datasets used in this study, the programs used to train and evaluate AdaBoost and Doc2Vec models, and the scripts to reproduce this study are available on Zenodo [55].
