# Supplementary material for: SonicParanoid2: fast, accurate, and comprehensive orthology inference with machine learning and language models
Source: Genome Biol. 2024 Jul 25;25:195. doi: 10.1186/s13059-024-03298-4 (PMC11270883; doi:10.1186/s13059-024-03298-4)
Supplement: Supplementary file 1 — Additional file 1: Supplementary text, figures (Figs. S1-S23), and tables (Tables S1-S12). [file 13059_2024_3298_MOESM1_ESM.pdf]

Additional File 1

Supplementary materials for:

SonicParanoid2: fast, accurate, and comprehensive orthology inference with machine learning and language models

Authors: Salvatore Cosentino[1], Sira Sriswasdi[2], Wataru Iwasaki[1,3-7]

Affiliations:

1. Department of Integrated Biosciences, Graduate School of Frontier Sciences, the University of Tokyo, Kashiwa, Japan

2. Center of Excellence in Computational Molecular Biology, Faculty of Medicine, Chulalongkorn University, Bangkok, Thailand

3. Department of Biological Sciences, Graduate School of Science, the University of Tokyo, Bunkyo-ku, Japan

4. Department of Computational Biology and Medical Sciences, Graduate School of Frontier Sciences, the University of Tokyo, Kashiwa, Japan

5. Atmosphere and Ocean Research Institute, the University of Tokyo, Kashiwa, Japan

6. Institute for Quantitative Biosciences, the University of Tokyo, Bunkyo-ku, Japan

7. Collaborative Research Institute for Innovative Microbiology, the University of Tokyo, Bunkyo-ku, Japan

Contact

Salvatore Cosentino (salvocos@k.u-tokyo.ac.jp)

Wataru Iwasaki (iwasaki@k.u-tokyo.ac.jp)

Supplementary Text:

Outline of SonicParanoid's graph-based algorithm.

SonicParanoid [31], described in Cosentino and Iwasaki (2019), predicts the ortholog genes between two species using a substantially modified algorithm from the approach used in the original InParanoid, where seed orthologs are identified using the bidirectional best hit (BBH). Next, the sequence similarities obtained for the seed orthologs are compared to those obtained for the intra-species alignments to identify candidate in-paralogs.
One difference with the original InParanoid is in the scoring function used to identify the in-paralogs. SonicParanoid uses a function that exponentially penalize candidate orthologs which length is considerably different from the orthologs obtained using the BBH.

$$f(a_p) = \begin{cases} \frac{score(a,a_p) - score(a,b)}{score(a,a) - score(a,b)}, & \text{if } d = 0 \\ \left(\frac{score(a,a_p) - score(a,b)}{score(a,a) - score(a,b)}\right)\left(1 - \left(\frac{3}{2}\right)^{\frac{ldr-1}{ldr}}\right), & \text{otherwise} \end{cases}$$

The candidate orthologs are then clustered using a greedy algorithm into clusters orthologs shared amongst pairs of species.
SonicParanoid also integrates the ability to predict orthogroups (orthologs shared amongst multiple species), and in recent versions it uses MCL instead of the original single linkage clustering.
Because SonicParanoid was published as an application note, which limits the length of the manuscript to only one page, the details cannot be found in the main text of the paper.
An extensive description of the SonicParanoid algorithm, as well as additional figures, can be found in the supplementary data of Cosentino and Iwasaki [31] (https://oup.silverchair-cdn.com/oup/backfile/Content_public/Journal/bioinformatics/35/1/10.1093_bioinformatics_bty631/3/bioinformatics_35_1_149_s1.pdf?Expires=1714522188&Signature=FwuT~W0zcKAezVxV3hK6Skim1HBoVjRzZ4R7Ew3PxyS6WS-nQCy8O89-xGDPjKJwXr7LxjxMsqR99F4mT91e-fTitMP8myRNq57DnzJpXgcOsSJz298nBxLIYuqF1fFUabZuijSG2a-kWOafLK92X2SK1645zFT~R7v7nbpYeoQXdIvCFcR0j8M1bPUdfFyEYPG3c9vesk61GJCvpIFti5z5iMHje6mtzcN7z6DJzsrNfISX2hgTIj9cTmYVO-5CKhgqS0zVaFpVArsRwMGZ1Hq0DLOIAgrwrJ5FDjeuq6At58sKI8b3Fovg1ioPzXkU~0WVNqk0H4biv8UGCLNcaQ__&Key-Pair-Id=APKAIE5G5CRDK6RD3PGA).

CPU-time, wall-time, and memory estimation

CPU-time and wall-time for each run shown in this study were computed using hyperfine (https://github.com/sharkdp/hyperfine). We set the "—runs" parameter to 1 to instruct hyperfine to perform a single a run. The JSON files generated by hypefine are stored in https://gitlab.com/salvo981/sonic-manuscript/genome_biology_review_files/round1/logs.

For each run the CPU-time is computed as the sum of *user* and *system* time.
Following is the template command used for hypefine:
hyperfine --style none --runs 1 –export-json hyperfine.results.json "<command to run>".
<command to run> can be any of the runs using any of the tested tools (e.g. SonicParanoid).
Memory estimation was performed using memory_profiler.py (https://pypi.org/project/memory-profiler/). In order to let the tool take into account the memory shared by the forked processes (or child processes), we use the psutil_pss backend instead of the deafult (psutil). Using psutil would simply perform a cumulative sum of the memory that forked job access, even when it is shared. This might lead to not realistic

memory peak estimations.
mprof run --nopython --backend psutil_pss --multiprocess -C –output memory_usage.dat <command to run>. We also use the “peak” sub-command to extract the memory usage peaks from the .dat files.
All the files, including the *.dat* files with the estimated memory peaks are stored under
https://gitlab.com/salvo981/sonic-manuscript/genome_biology_review_files/round1/logs/memory-profiling-using-qfo/

Method ranking using classification methods from the QfO benchmarks
The ranks shown in Fig 2e are generated by aggregating the counts of the groups for given by the three classification methods for each participant in the benchmark.
The bash scripts to generate the ranking plots included in this manuscript can be found at
https://gitlab.com/salvo981/sonic-manuscript/plots/qfo-benchmarks-ranking
While the data-points can be found under
https://gitlab.com/salvo981/sonic-manuscript/datapoints/manuscript/domain-orthology/qfo20-webpage-method-ranking/qfo20-webpage-method-ranking.tar.xz
These data-points were obtained by counting, for each method, and for each test how many times it is classified as belonging to 1st, 2nd, 3th, or 4th group.
The table from which this information were obtained is publicly available at the QfO benchmark page.
https://orthology.benchmarkservice.org/proxy/results/2020/#tab_generalized-species-tree-discordance-benchmark_fungi_OEBD00200002AN
[just click on “Summary table” after you open the link]
The aggregate results, are obtained by summing the counts from the 3 classification methods.

The three classification methods were developed as part of OpenEBench pipeline, and the relative code is available at
https://github.com/inab/OpenEBench_scientific_visualizer
The above is the same code used in the QfO benchmark page
(http://orthology.benchmarkservice.org/)
The script to perform the clustering is
https://github.com/inab/OpenEBench_scientific_visualizer/blob/master/src/classification.js
while the quartiles are generated using the d3’s square quartile method, while the clustering uses the “cluster” package from
https://www.npmjs.com/package/clusters

Ability of the compared tools to process big datasets
While, all tools can process the QfO dataset easily, only SonicParanoid2 and ProteinOrtho6 were able to process the 2,000 MAGs dataset. It should be noted that ProteinOrho6 writes a great amount of small files which, when processing big datasets (e.g. >100), results in a very high amount of I/O operation which ultimately slow down the system.
Broccoli, used way too much memory (>2TeraBytes in our case) and caused the server to SWAP when processing both the 200 eukaryotes and 2,000 MAGs datasets.
OrthoFinder2 was able to predict the orthologs for the 200 Eukaryote dataset but required manual intervention in order to run.
The problem is related to the tool opening N^2 files, and is described in the following issue:
https://github.com/davidemms/OrthoFinder/issues/571
We were not allowed to further increase the file limit required to process the dataset with 2,000 MAGs.

ProteinOrho6 failed to process sequences which contain the translation stop symbol “*”. Because of this, we modified the 2,000 MAGs dataset to remove the “*” in order to run ProteinOrtho.
The software failed with the following error:

```
[1;31m[Error][0m [1;33m
ERROR found line with forbidden symbols in
'/ssd_home/sp2-genome-biology-review-runs/input/reference_2000_mags/2156126005_1.faa' that is
'*' (/^[^a-z#>]/i)
full line:
*

 [0m
```

In order to help the developers, we also opened an issue in the GitLab page of ProteinOrho6.
https://gitlab.com/paulklemm_PHD/proteinortho/-/issues/91

Data sources for the proteomes
We have provided the tables listing the UniProt proteome IDs for the 200 eukaryotes dataset, which was used to evaluate the tools, and the 250 eukaryotes-prokaryotes dataset, which was used to train the AdaBoost model for selecting the faster inter-proteome alignment to perform, as Additional File 2: Table S13 and Additional File 3: S14, respectively.

Supplementary Figures:

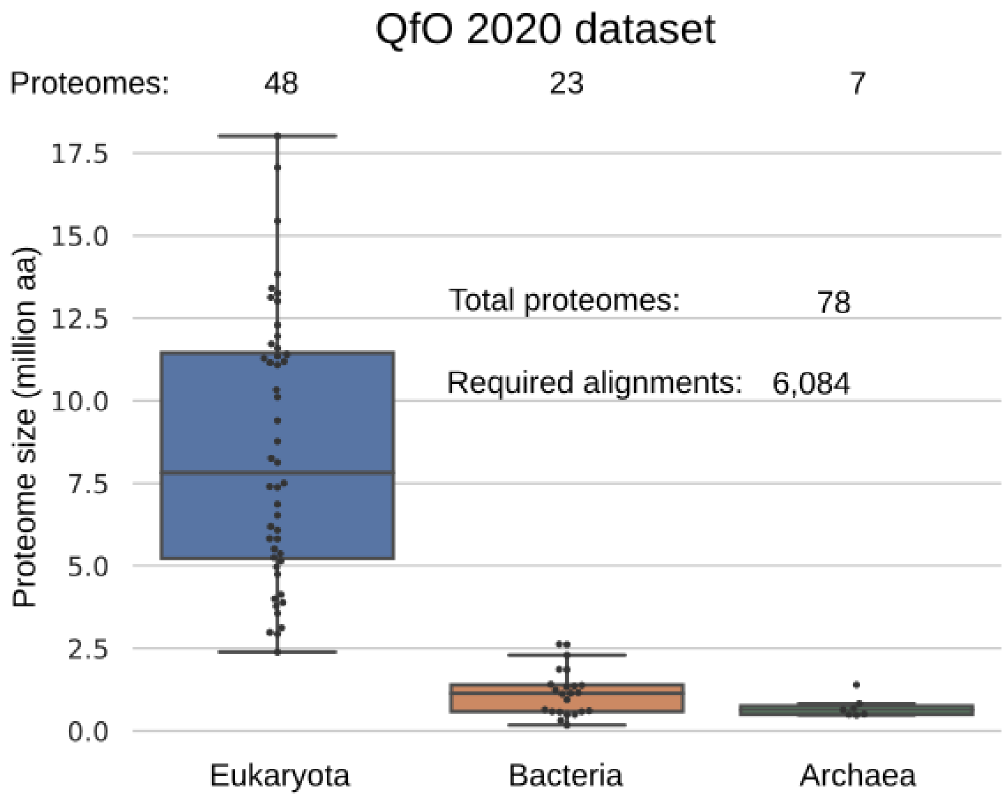

Fig S1: QfO 2020 test datasets. Benchmark dataset from the QfO consortium. The dataset comprises 78 proteomes with an average size of 5.63 million AA.

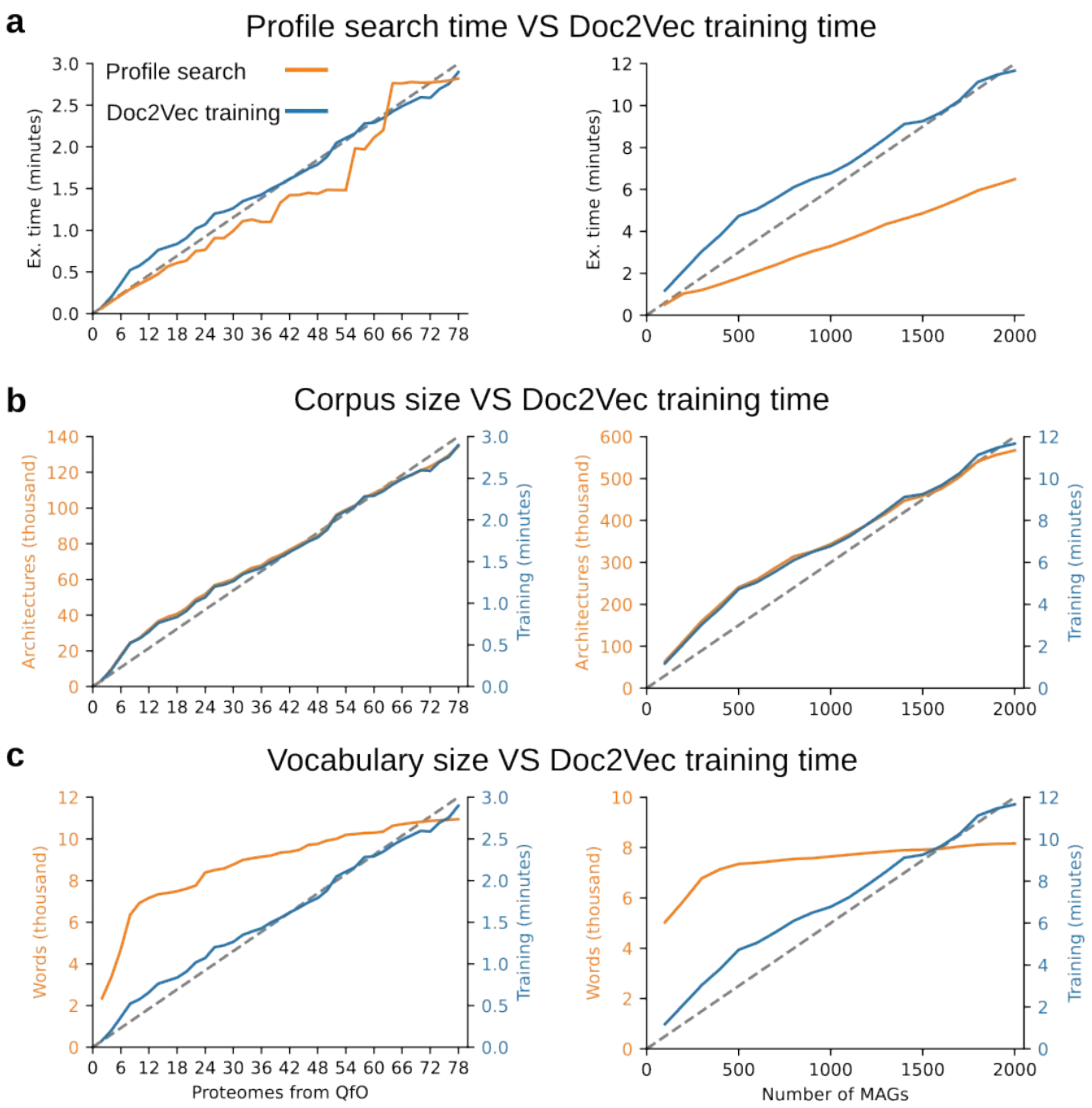

Fig S2: Scalability of domain-based pipeline. Plots on the right and left refer to trials with QfO and MAG datasets as input, respectively. Multiple trials were performed using the domain-based pipeline with increasing numbers of input proteomes from both datasets (x-axis). Domain-based pipeline shows high scalability with a quasi-linear execution time growth as the number of input proteomes increases. **a**, Increase in execution time for profile searches and Doc2Vec model training. Total execution time for both steps was approximately 6 min for the complete QfO dataset and approximately 18 min (only 3X longer) for the 2,000 MAGs. **b**, Time required to train the Doc2Vec models increases linearly with the corpus size. **c**, Size of vocabulary (single words in the corpus) reaches a plateau when the input proteomes have similar contents (e.g. for the MAG dataset).

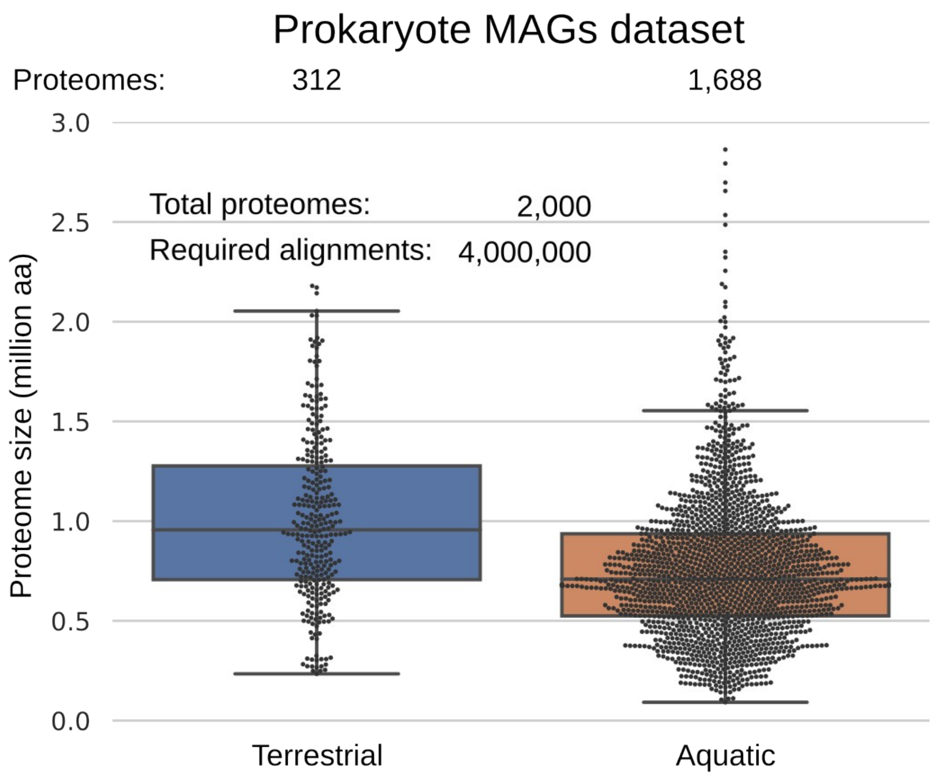

Fig S3: 2,000 MAGs dataset. Bacterial MAGs from terrestrial and aquatic environments randomly selected from a catalogue of 52,515 MAGs described by Nayfach et al. 2021.

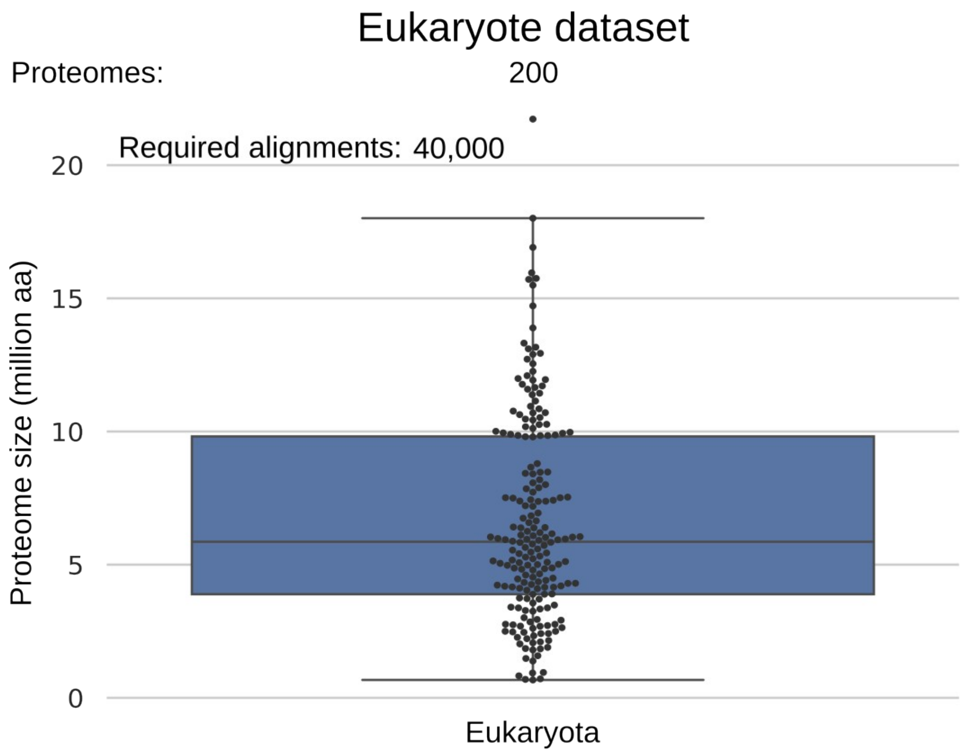

Fig S4: 200 Eukaryote dataset. The files in the dataset are all reference proteomes obtained from Uniprot.

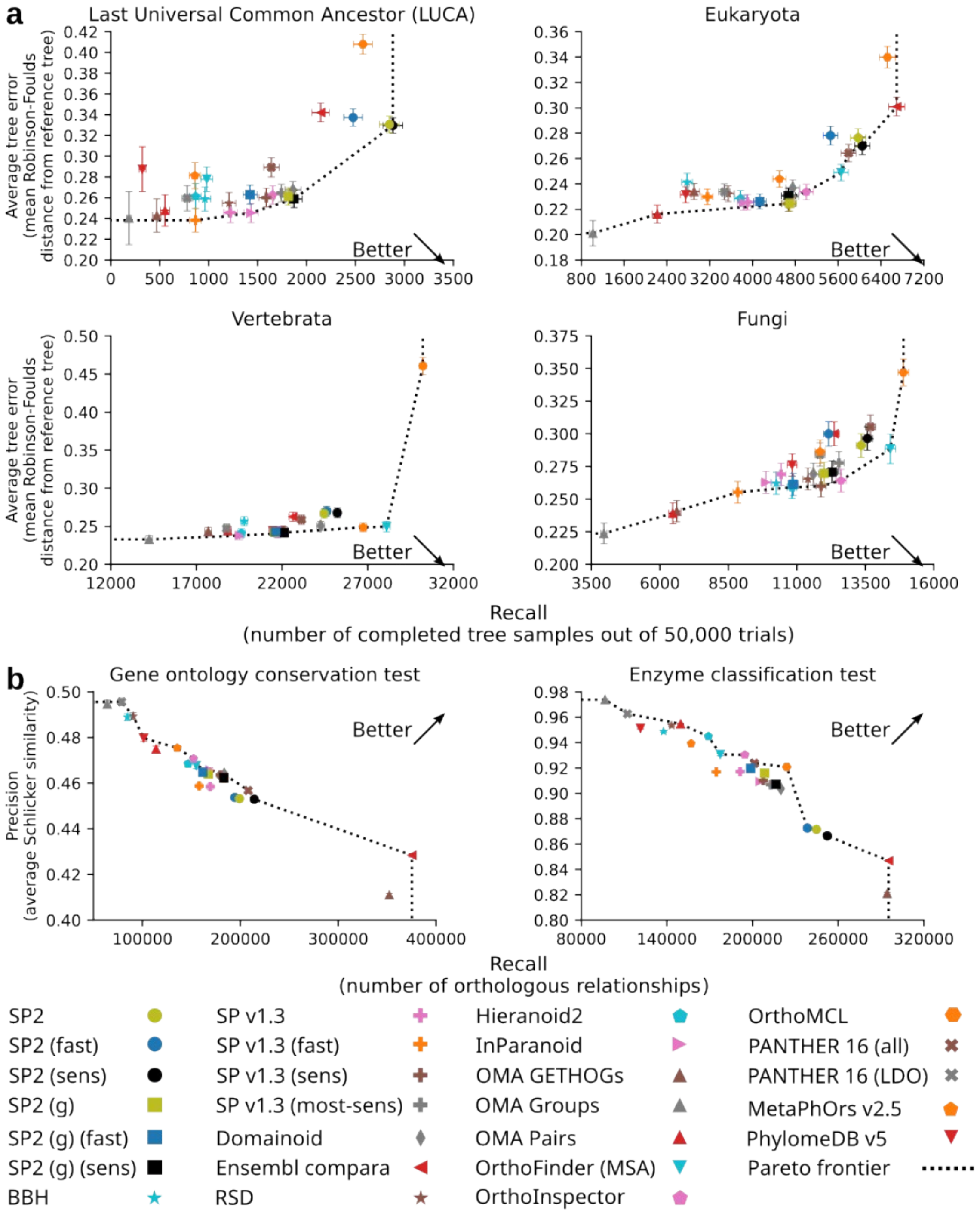

Fig S5: Accuracy of SonicParanoid2 with and without domain-based orthology. SP2 runs denoted with "g" were performed using the graph-based algorithm. Methods shown by markers on the Pareto frontier demonstrate the best balance between precision and recall. Square markers represent accuracies obtained when using only the graph-based pipeline. Results of **a**, generalized species tree discordance tests and **b**, functional benchmark tests.

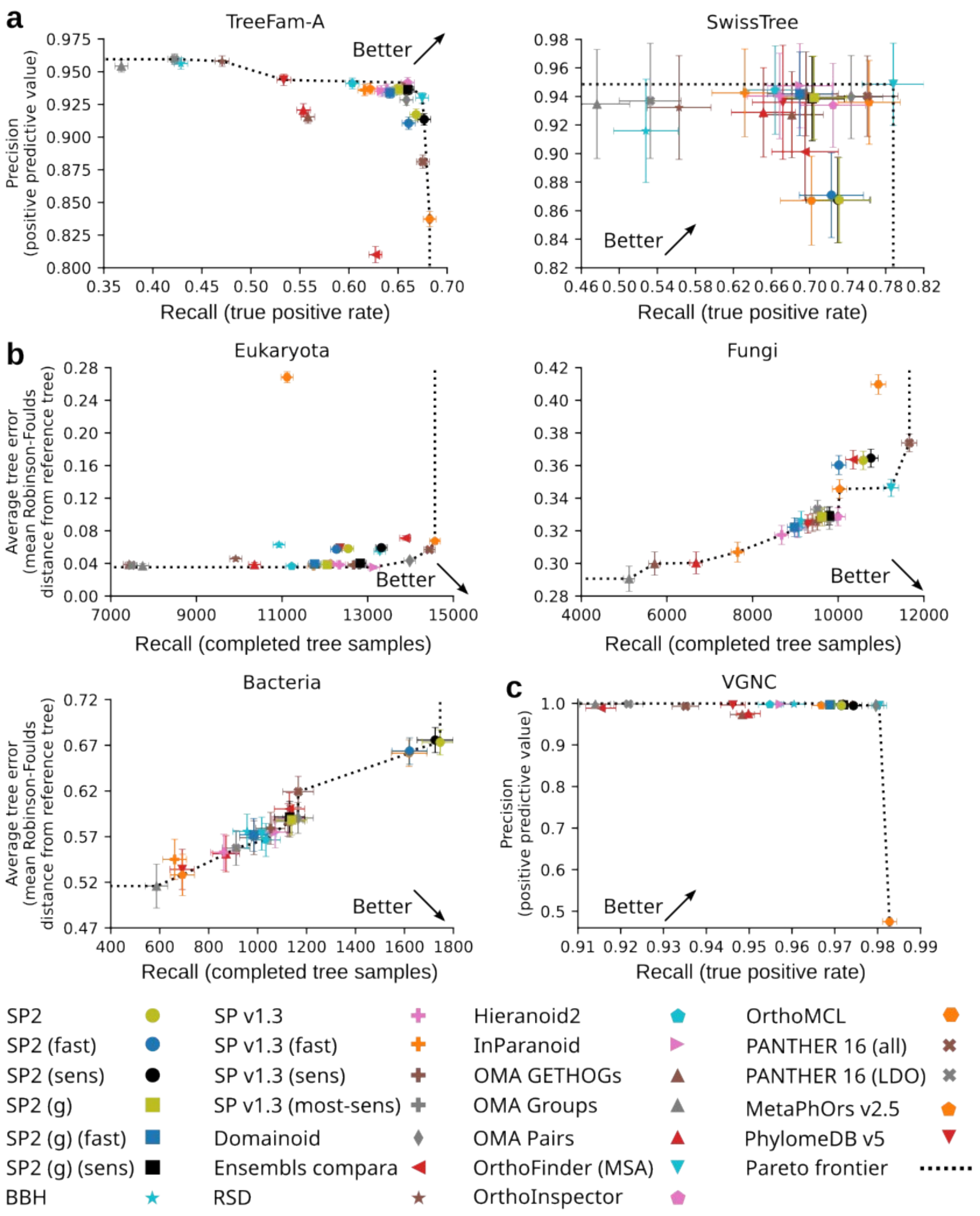

Fig S6: Accuracy of SonicParanoid2 with and without domain-based orthology. SP2 runs denoted with “g” were performed using the graph-based algorithm. Methods shown by markers on Pareto frontier demonstrate the best balance between precision and recall. Square markers represent accuracies obtained when using only the graph-based pipeline. Results of **a**, reference-tree based tests, **b**, species tree discordance tests (eukaryota, fungi, bacteria), and **c**, Vertebrate Gene Nomenclature Committee (VGNC) test.

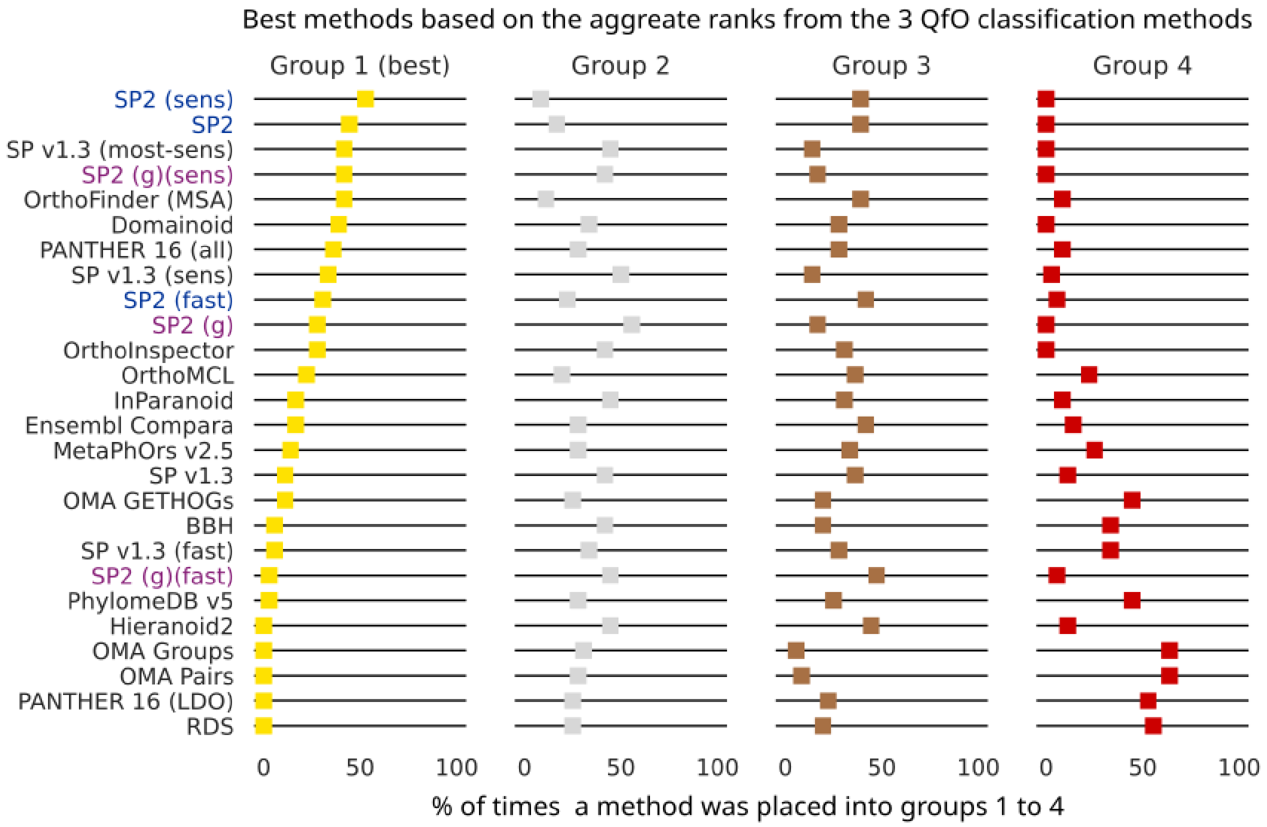

Fig S7: Accuracy of SonicParanoid2 and other 14 methods based on the aggregate ranking from the three classification methods of the QfO benchmark. For each test in the benchmark suite, a method was assigned to group 1 if it had very high precision and recall (e.g., close to "optimal performance" corner), and group 4 if it was close to the "suboptimal performance" corner. Based on a total of 36 rankings (12 from each classification method) participants were sorted on the percentages of tests they were assigned to each group. Blue labels represent SonicParanoid2 executions which included the domain-based orthology inference, whereas purple labels refer to runs in which only graph-based orthology was performed.

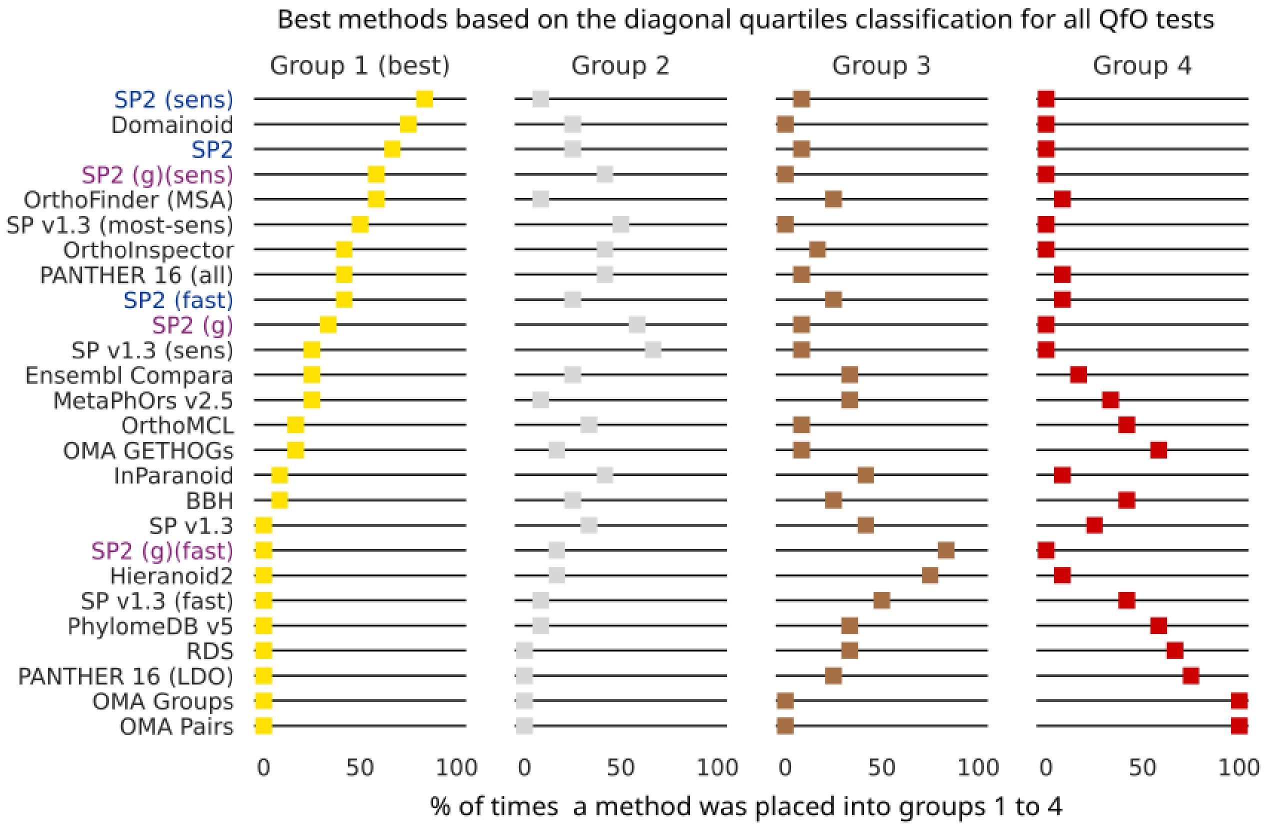

Fig S8: Accuracy of SonicParanoid2 and other 14 methods based on diagonal quartiles classification from the QfO benchmark. For each of the 12 tests, participants were assigned a group based on their distance from the “optimal perfomance” corner. Methods were sorted on the percentages of tests they were assigned to each group. Blue labels represent SonicParanoid2 executions which included the domain-based orthology inference, whereas purple labels refer to runs in which only graph-based orthology was performed.

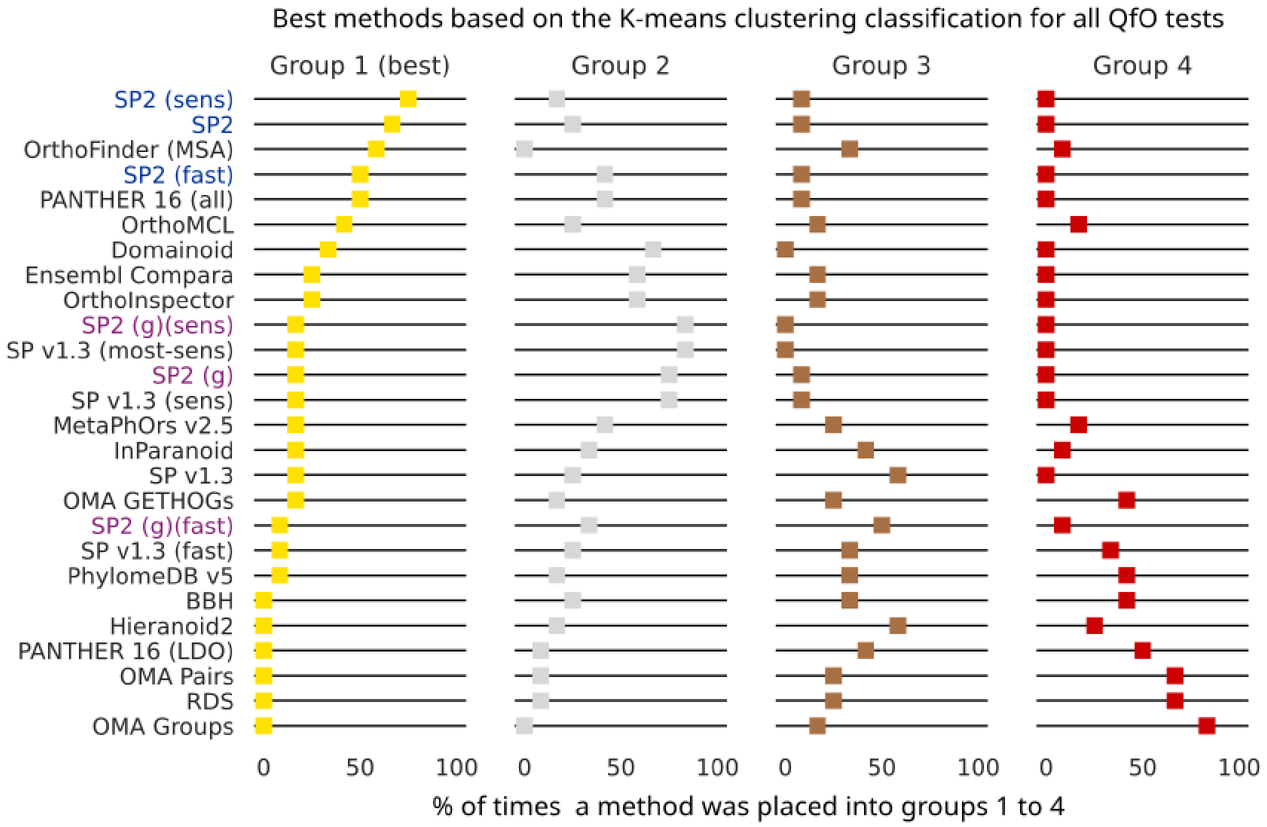

Fig S9: Accuracy of SonicParanoid2 and other 14 methods based on K-means clustering classification from the QfO benchmark. For each of the 12 tests, participants were separated into 4 clusters (groups) using the K-means algorithm. Methods were sorted on the percentages of tests they were assigned to each group. Blue labels represent SonicParanoid2 executions which included the domain-based orthology inference, whereas purple labels refer to runs in which only graph-based orthology was performed.

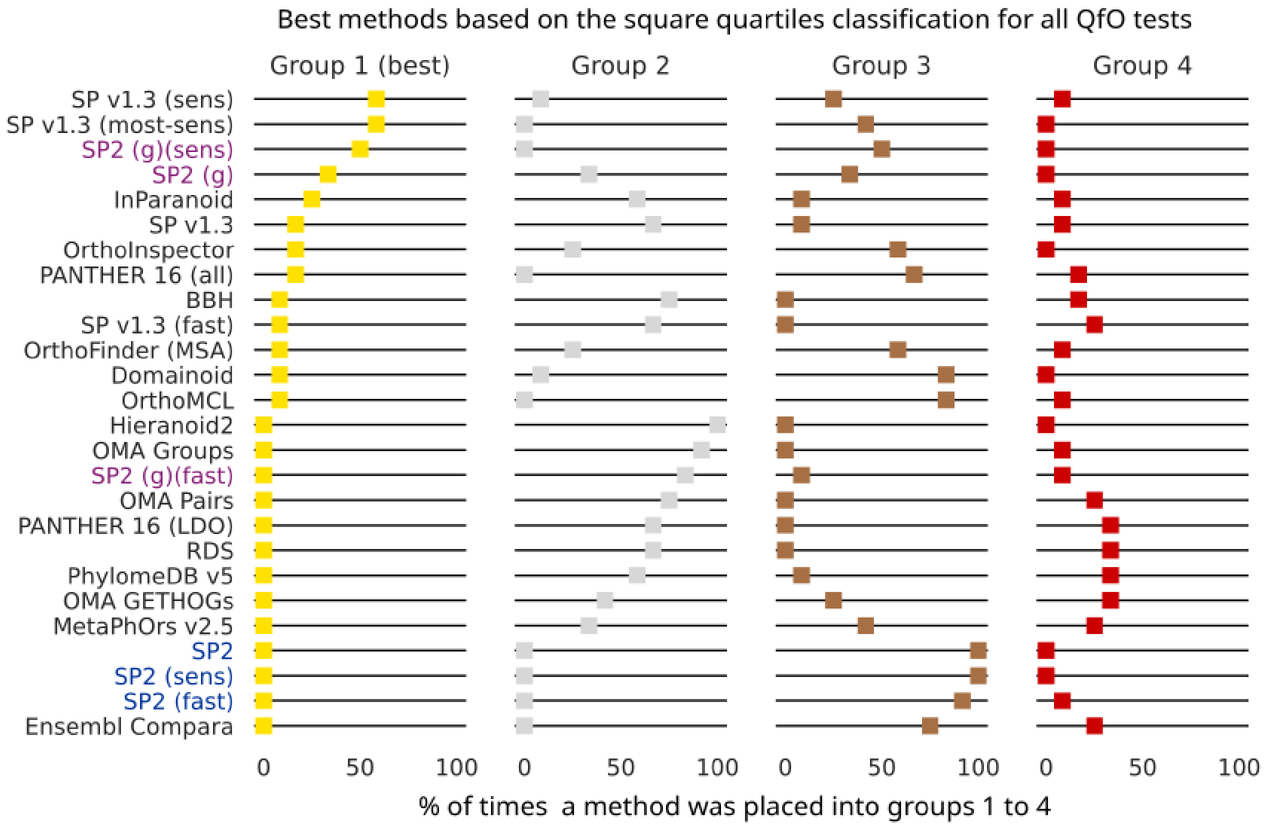

Fig S10: Accuracy of SonicParanoid2 and other 14 methods based on the square quartiles classification from the QfO benchmark. For each of the 12 tests, participants were assigned a group by checking if they were above or below of the first quartiles of the precision and recall. Methods were sorted on the percentages of tests they were assigned to each group. Blue labels represent SonicParanoid2 executions which included the domain-based orthology inference, whereas purple labels refer to runs in which only graph-based orthology was performed.

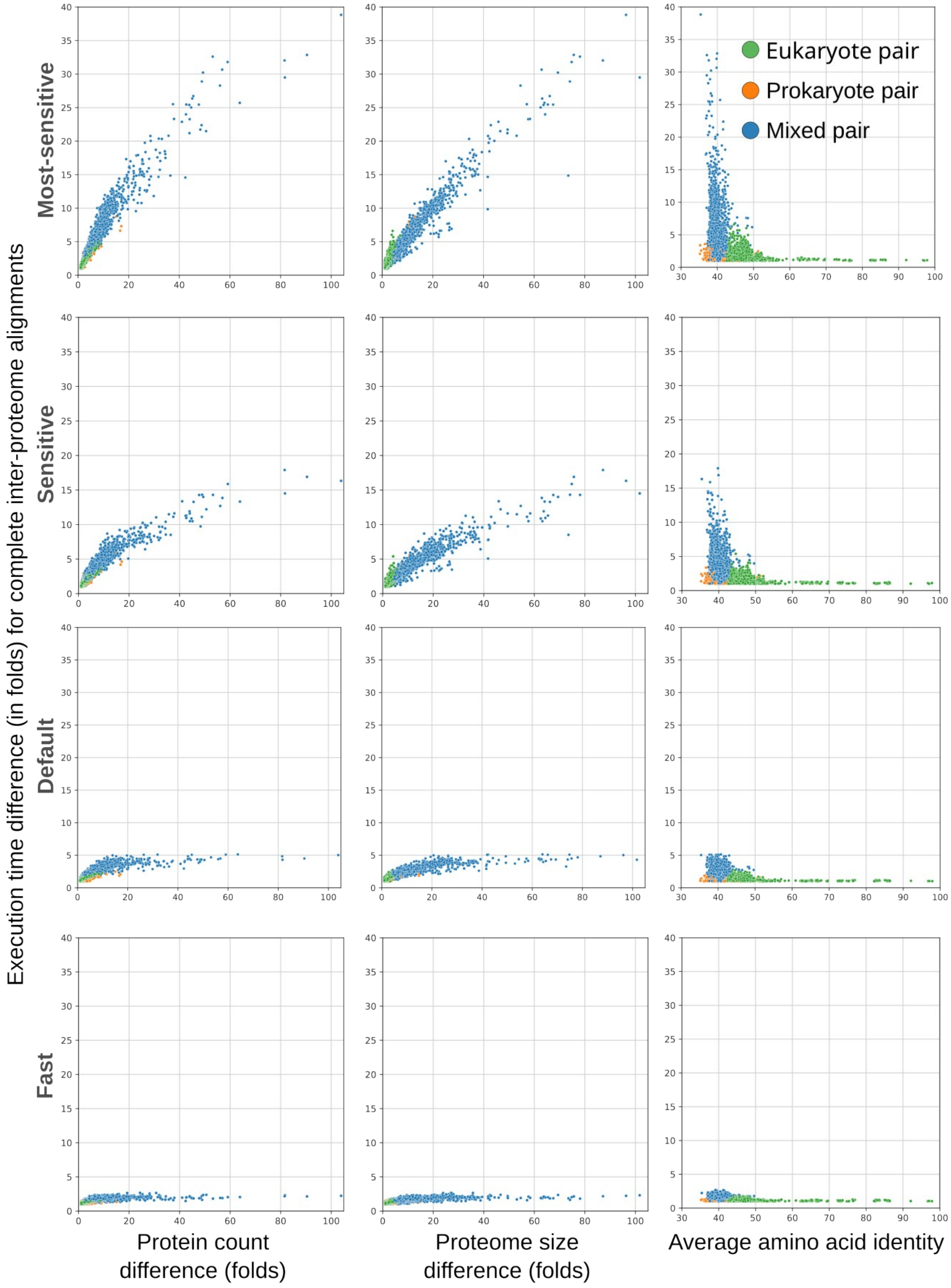

Fig S11: Execution time differences between complete inter-proteome alignments using MMseqs2 at multiple settings. The execution time difference for a specified pair (e.g., A-B) is computed as the absolute value of the execution times of the two inter-proteome alignments. Each dot represents one of the 3,003 possible combinations for the QfO dataset.

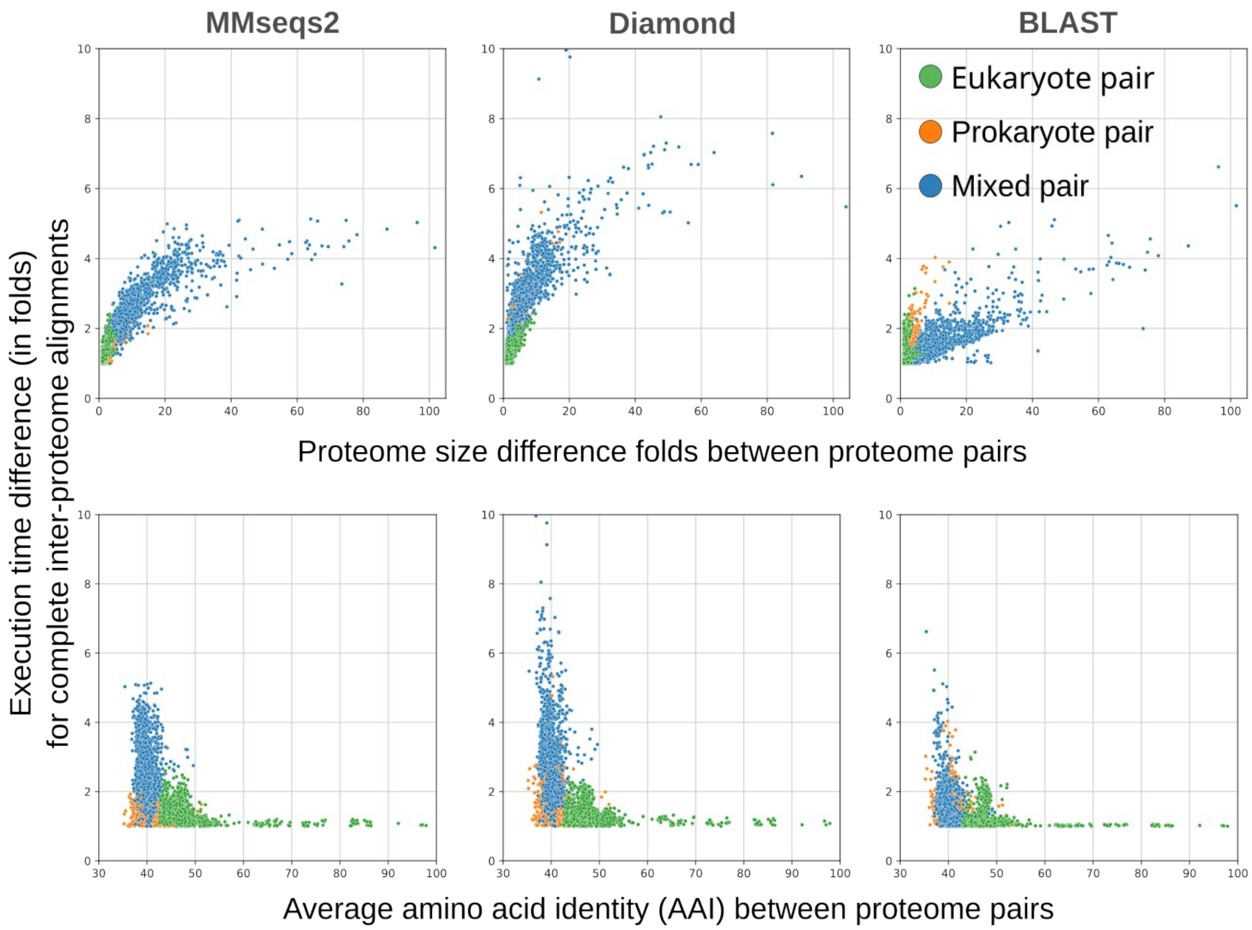

Fig S12: Execution time differences between complete inter-proteome alignments using multiple local alignment tools. Y-axis shows execution time difference between inter-proteome alignments for each of the 3,003 possible combinations in the QfO proteome set, using (left to right) MMseqs2, Diamond, and BLAST at the default mode (as in Supplementary Table S3). As in Figure S9, the execution time difference is proportional to the difference in proteome size (top row) and inversely proportional to AAI (bottom row).

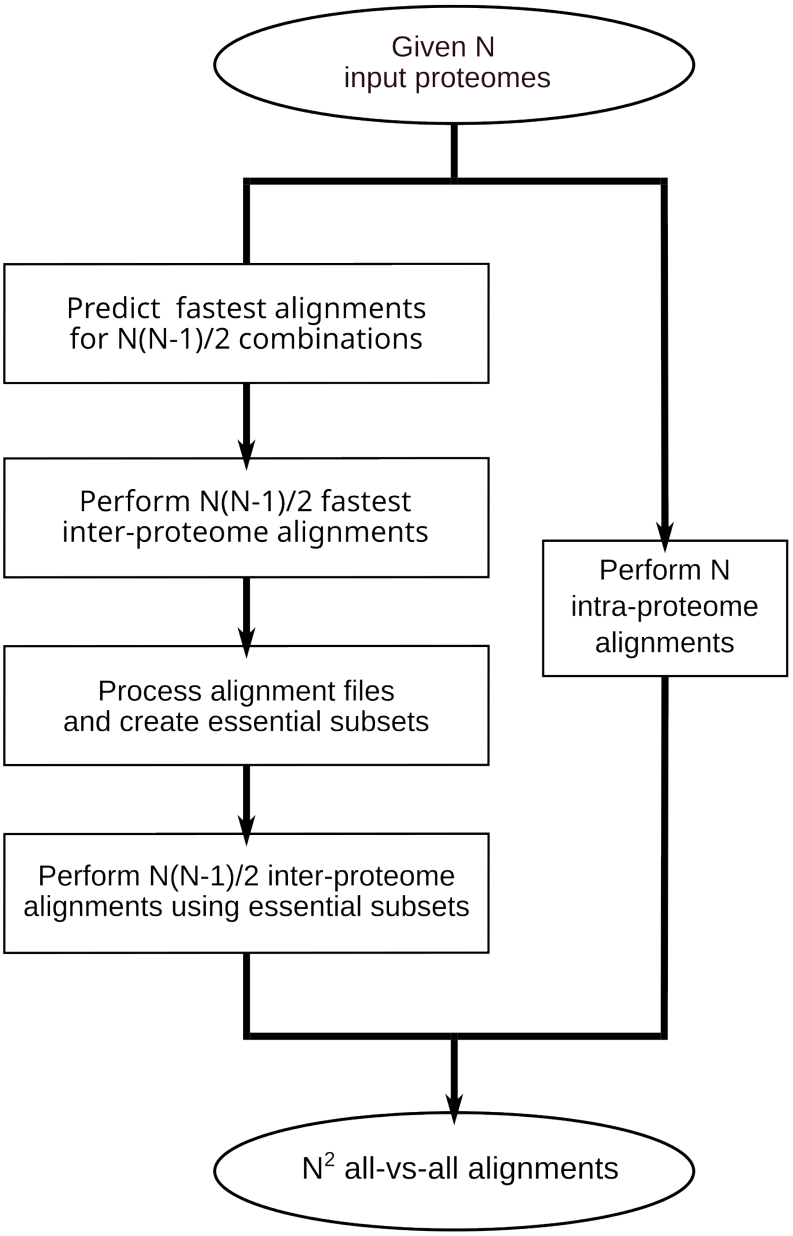

Fig S13: Flowchart of AdaBoost-enhanced graph-based method in SonicParanoid2. For each possible combination of *N* input proteomes, the fastest inter-proteome alignments are predicted using an AdaBoost binary classifier (left branch). After the fastest alignments are performed, they are processed and the essential protein sets are generated, as in Equation 2. The remaining inter-proteome alignments are performed on the reduced protein sets. The intra-proteome (right branch) and the predicted fastest inter-proteome alignments are performed using the complete input proteomes.

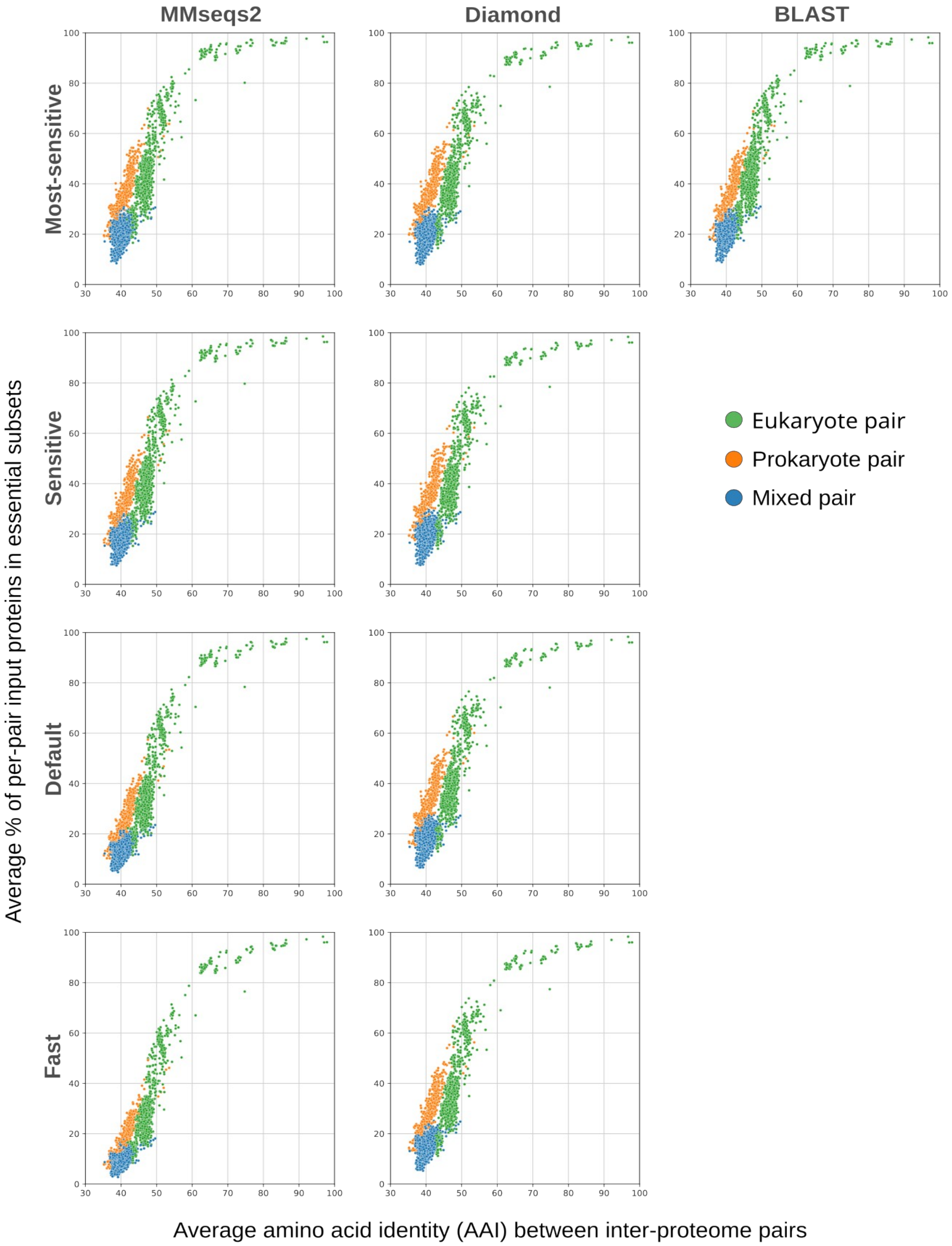

Fig S14: Percentage of input sequences used and evolutionary relatedness in graph-based orthology inference (QfO 2020). Y-axis shows the average percentage of original input constituting the essential subsets of each pair of proteomes. For example, if the essential sets for A and B contain 15% and 25% of the original input proteins, respectively, then 20 will be on the y-axis. The percentage of the original input proteins used is directly proportional to the evolutionary relatedness (expressed in terms of the AAI on the x-axis) and agnostic on the alignment tool used.

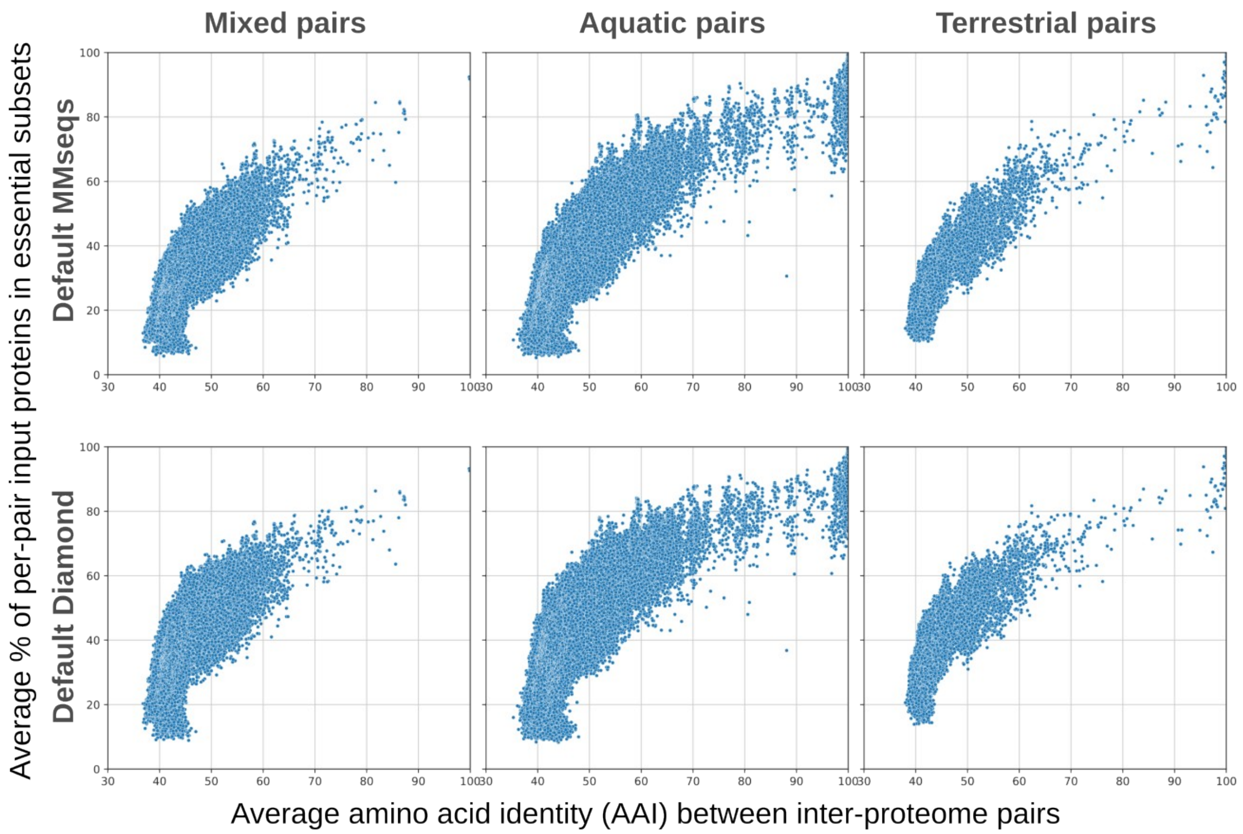

Fig S15: **Percentage of input sequences used and evolutionary relatedness in graph-based orthology inference (2,000 bacterial MAGs).** Y-axis shows average percentage of input proteins constituting the essential subsets of each inter-proteome alignment. Similarly to the observations for the QfO dataset (Supplementary Figure S12), the average percentage of input proteins used in the essential subsets is directly proportional to the evolutionary relatedness (expressed as AAI). The leftmost plots refer to pairs of MAGs, in which one MAG was obtained from aquatic samples and the other from terrestrial samples.

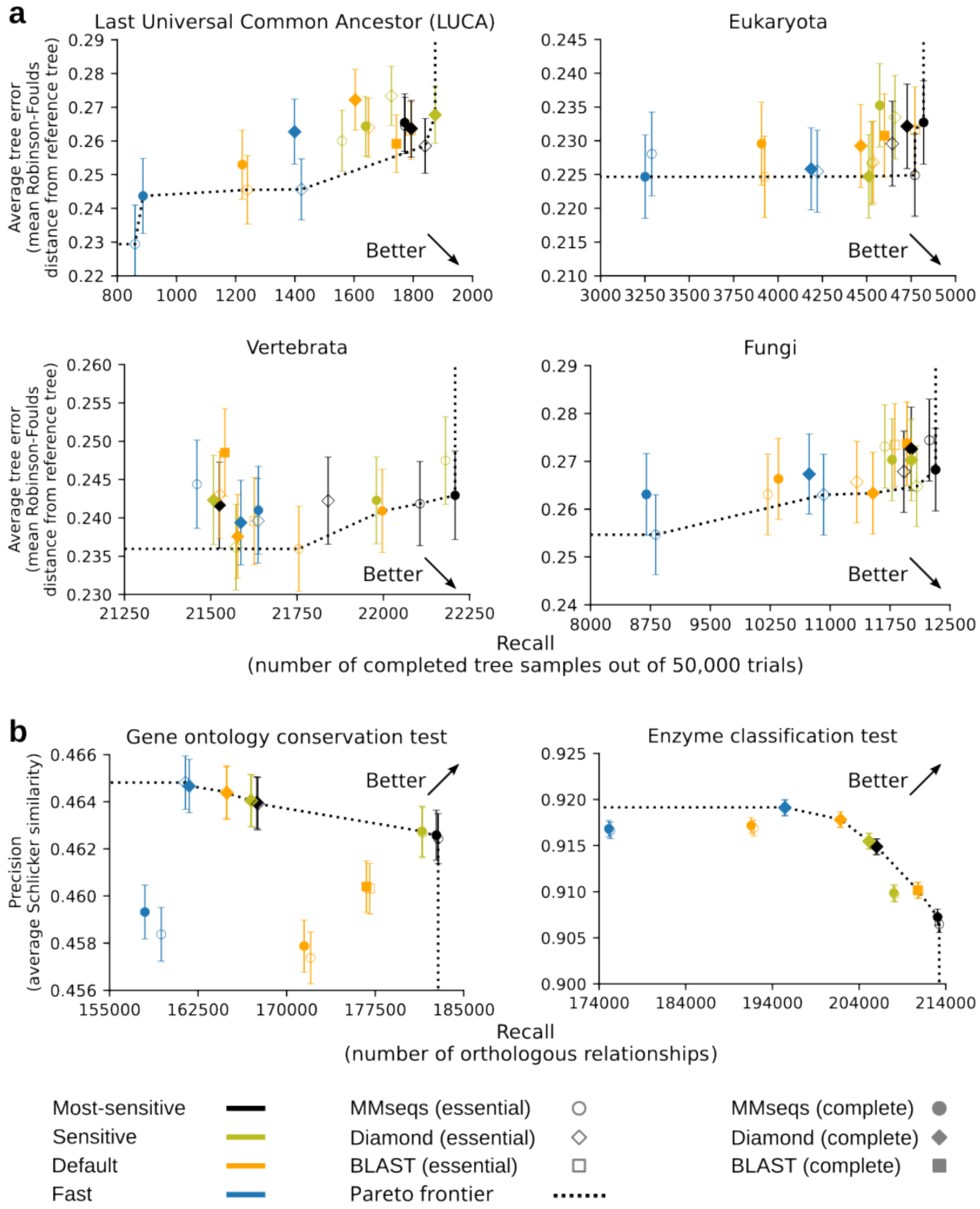

Fig S16: Benchmark results for predictions obtained with and without essential alignments. a, Accuracies of SonicParanoid2 obtained using essential alignments (empty markers) on the generalized species tree discordance tests are highly similar to those obtained using the complete alignments, regardless of the alignment tool and sensitivity settings used. **b**, The prediction accuracies on functional benchmark tests conducted using the essential and complete alignments are extremely similar and do overlap in many cases.

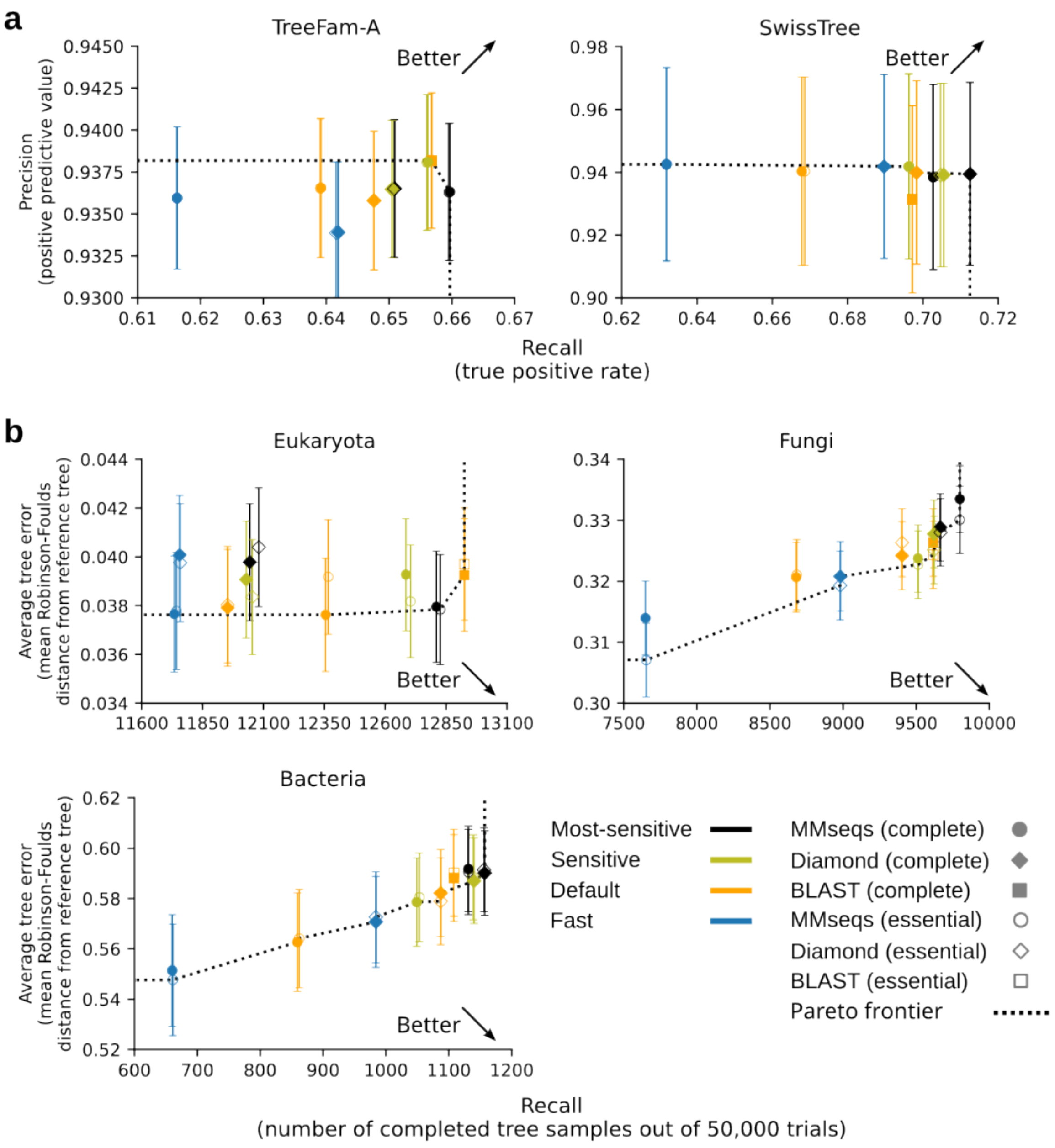

Fig S17: Benchmark results for predictions obtained with and without essential alignments. Empty markers represent predictions obtained using essential alignments. **a**, Prediction accuracies on gene-tree-based benchmark tests tend to overlap, which is likely due to the small sizes of the benchmark sets. Accuracies overlap on species tree discordance tests in **b** for fungi and bacteria, which use relatively small test sets. Accuracies based on eukaryote dataset appear more similar at higher sensitivity settings.

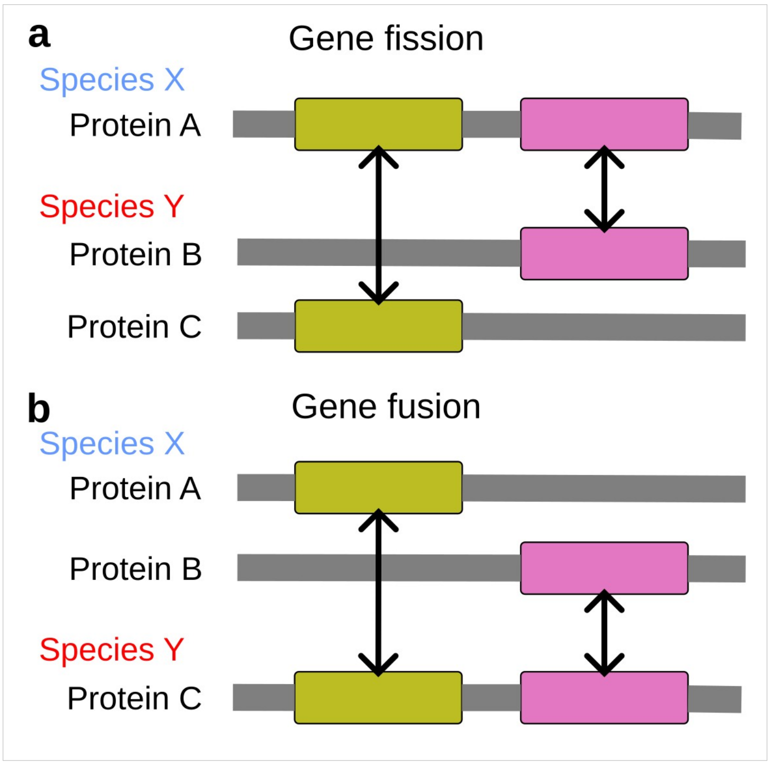

Fig S18: Orthologous domains typically missed by BBH. Proteins involved in fission or fusion events during evolution may contain orthologous relationships at the domain level (black arrows) that may be missed by BBH. **a**, Gene fission event in which a multi-domain protein from species X has its orthologous domains separated into two single-domain proteins in species Y. **b**, Gene-fusion event in which two domains in two different proteins in species X have their orthologs arranged into a single protein in species Y.

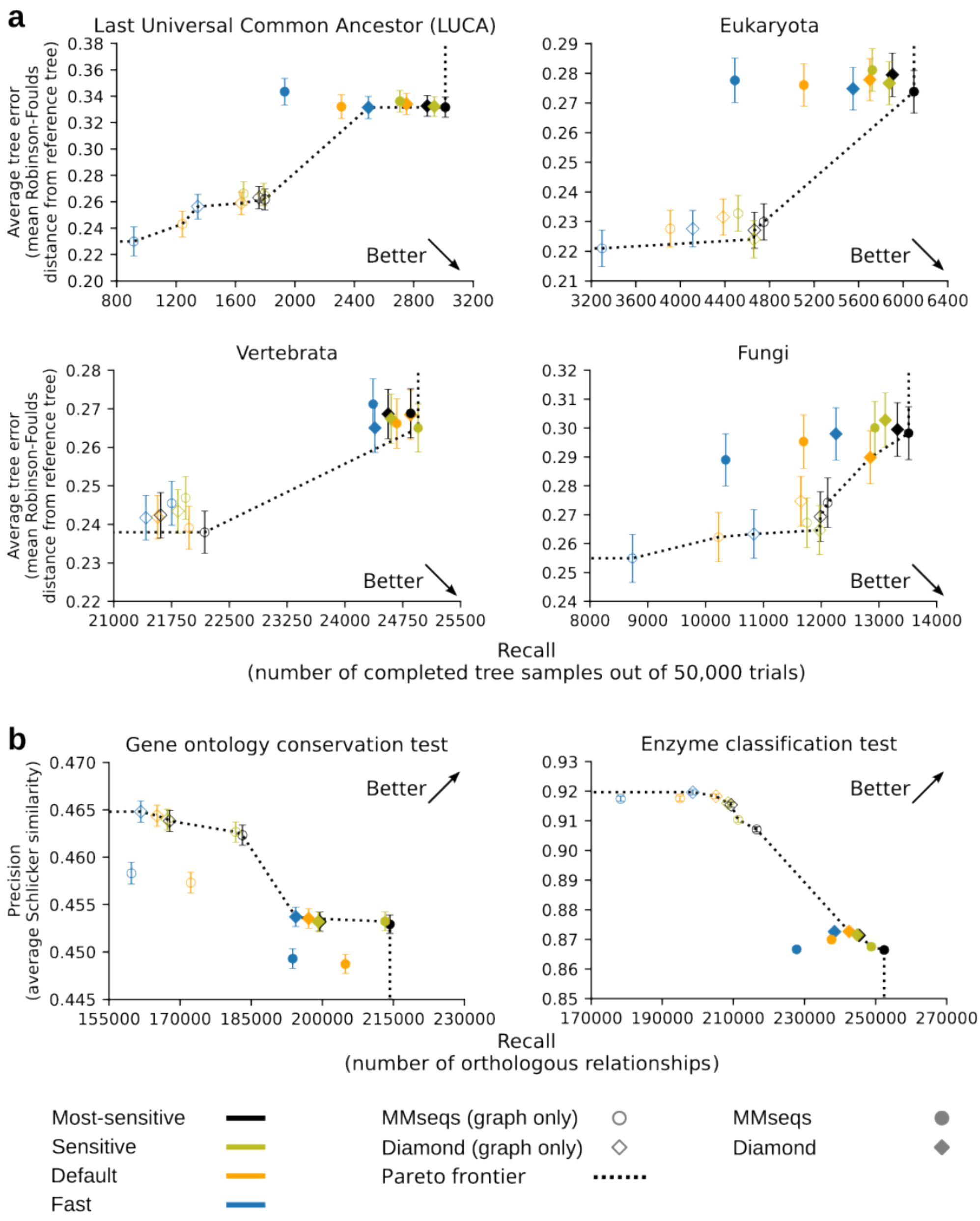

Fig S19: QfO benchmark results for SonicParanoid2 with and without domain-based orthology inference. Results shown by empty markers were obtained using the graph-based pipeline only (Figure 1a), whereas those shown by filled markers include orthologs predicted from the domain-based algorithm (Figure 1b). Settings described in Supplementary Table S3 were used. **a**, Accuracy results obtained from generalized species tree discordance tests. **b**, Prediction accuracies obtained from functional benchmark tests.

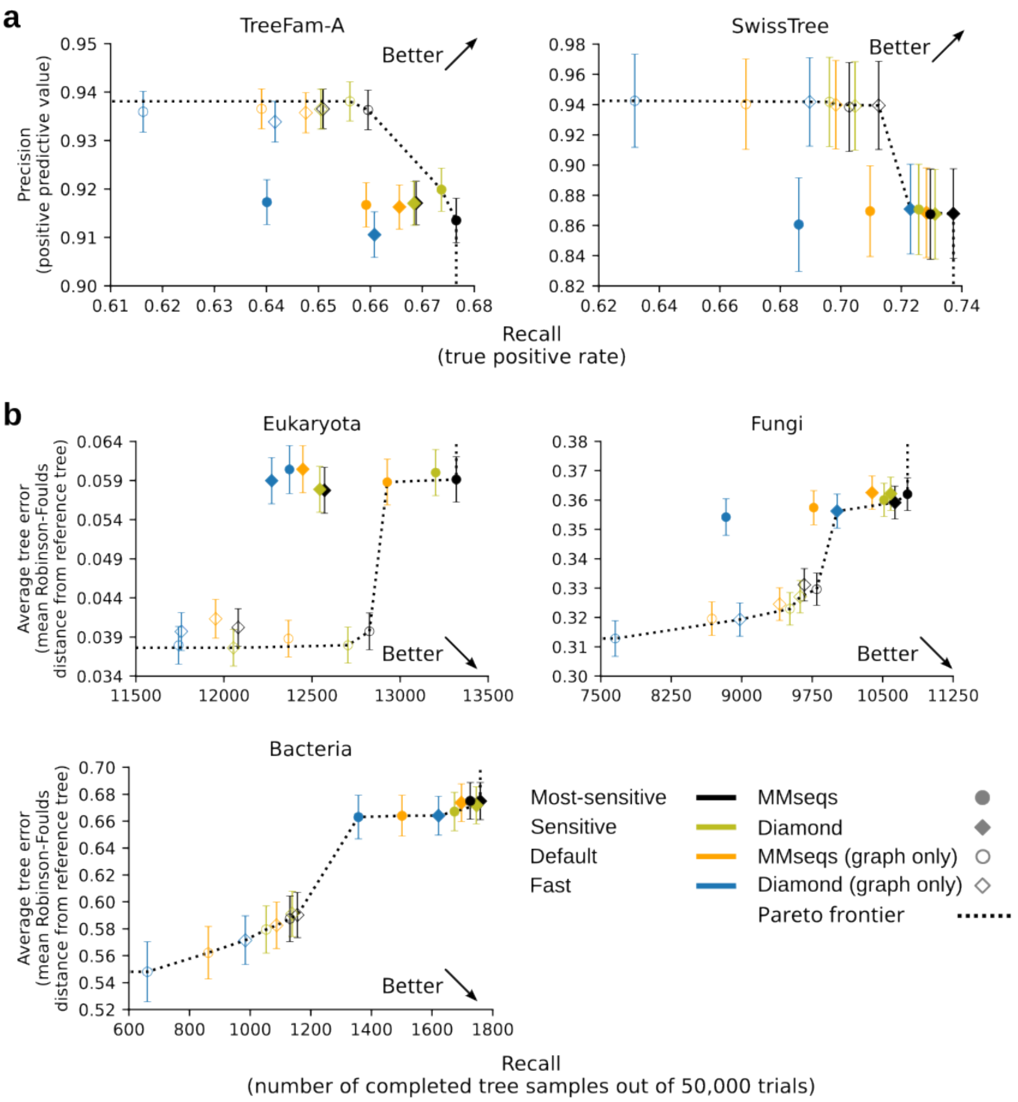

Fig S20: QfO benchmark results for SonicParanoid2 with and without domain-based orthology inference. Results shown by empty markers were obtained using the graph-based pipeline only (Figure 1a), whereas those shown by filled markers include orthologs predicted from the domain-based algorithm (Figure 1b). Settings described in Supplementary Table S3 were used. Results obtained from **a**, gene tree-based tests and **b**, species tree discordance tests.

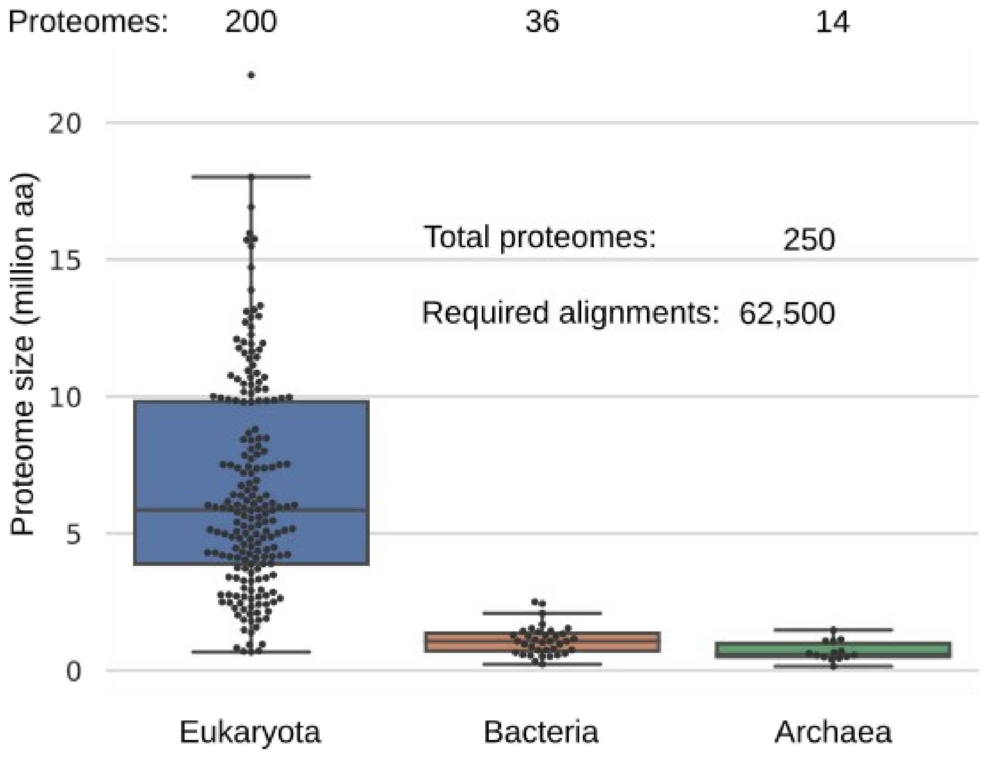

Fig S21: Dataset used to generate training samples and labels for binary AdaBoost classifier. Average size of the proteomes is 5.50 million AA, with *Glicine max* (21.73 million AA) and *Nanoarchaeum equitans* (0.15 million AA) being the largest and smallest, respectively.

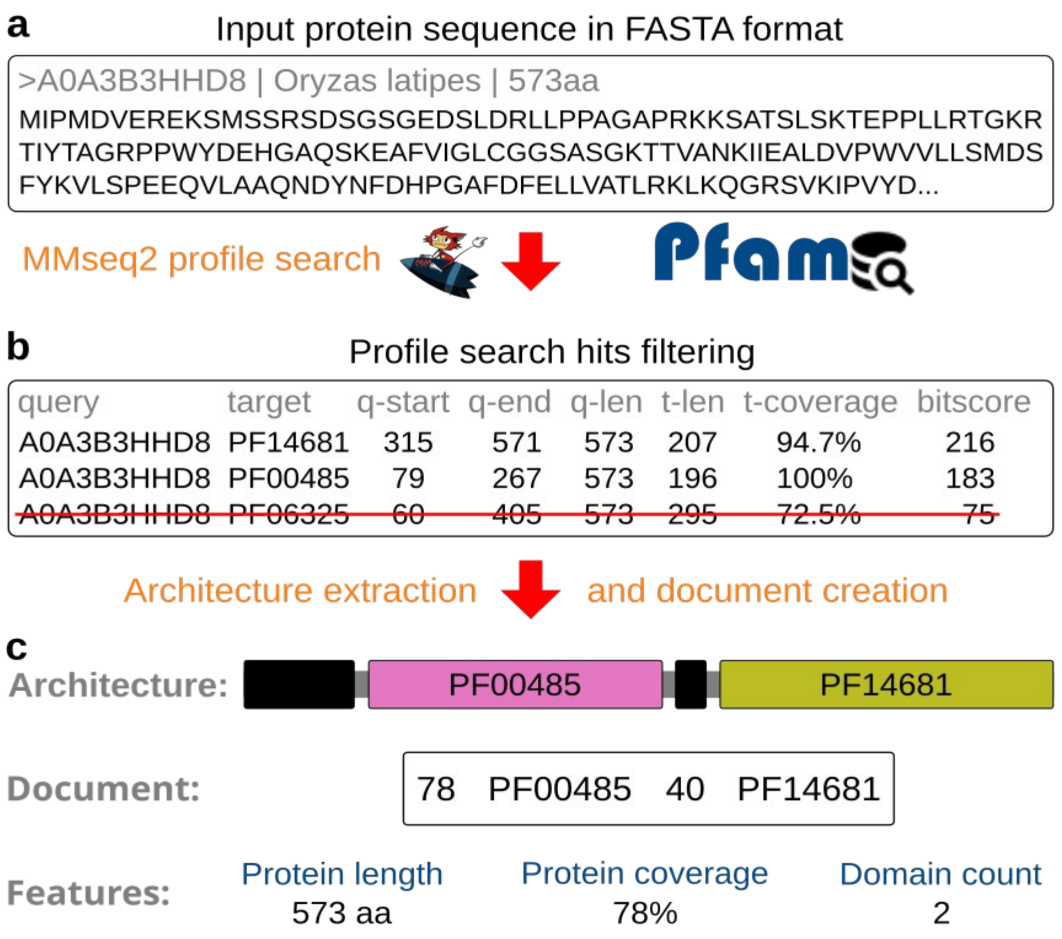

Fig S22: Example of architecture estimation and document creation for a single input protein. **a**, Search the protein in the Pfam profile database using MMseqs2. **b**, Only hits with a bitscore exceeding 30 and domain coverage (t-coverage) exceeding 75% are used to build the architectures. **c**, Representation of the architecture as a sequence of domains, document, and architecture features. Black blocks in the architecture represent unannotated regions.

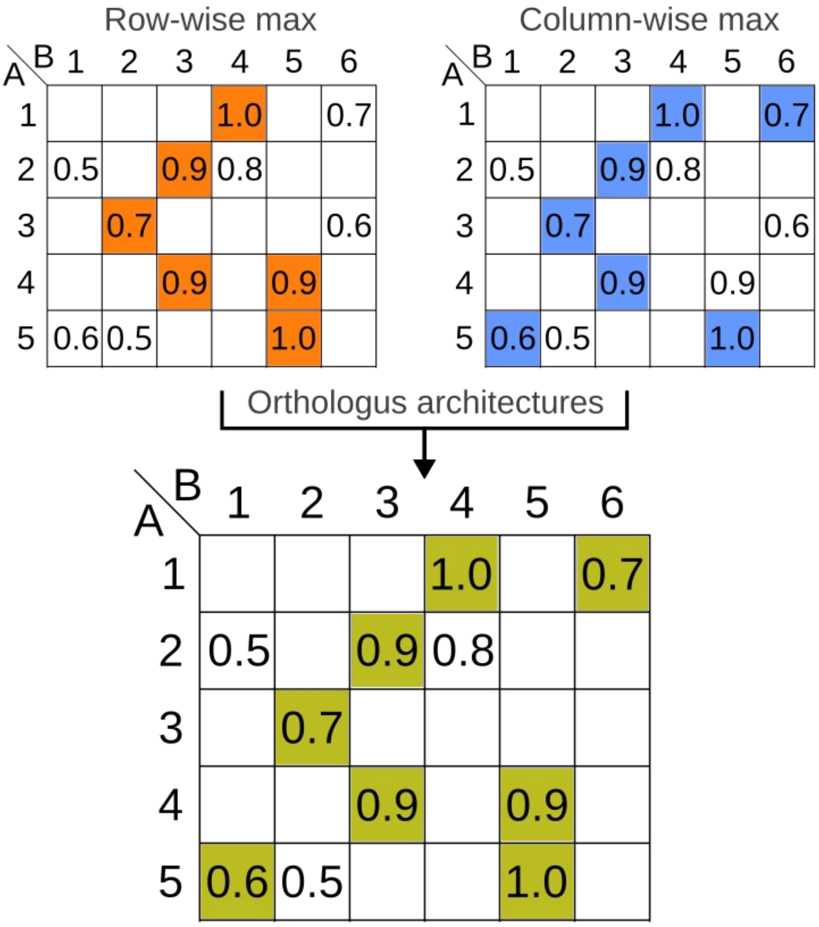

Fig S23: Selection of candidate orthologs in the domain-based pipeline. Maximum cosine similarities for each pair of architecture from proteomes A (5 proteins) and B (6 proteins) are selected. The pairs of architectures with maximum cosine similarity are selected row-wise (top-left) and column-wise (top-right). Pairs corresponding to entries in the final matrix (bottom) will populate the ortholog clusters generated by the domain-based pipeline.

Supplementary Tables:

SonicParanoid mode	Alignment tool	Alignment tool sensitivity settings
Default	Diamond	very-sensitive
Fast	Diamond	mid-sensitive
Sensitive	MMseqs	-s 7.5

Table S1: SonicParanoid2 presets. Alignment tools and settings used for the three SonicParanoid2 presets. In addition to the presets in the table, users can execute SonicParanoid2 using custom settings for the alignment tool (Diamond, MMseqs2 or BLAST) and its sensitivity.

Tool	Settings	Total (hours)	Speedup on ProteinOrtho	CPU time (hours)
SonicParanoid2	--diamond sensitive	0.696	1.006	52.674
SonicParanoid2	--diamond sensitive -go	0.509	1.374	40.477
ProteinOrtho 6	default	0.700	1.000	26.067

Table S2: SonicParanoid2 vs ProteinOrtho using the same sensitivity. SonicParanoid2 and ProteinOrtho execution times for QfO dataset when using the same sensitivity settings for Diamond. By default ProteinOrtho uses --diamond sensitive.

Input dataset: QfO 2020 (78 proteomes)					
**Tool**	**Settings**	**Extra Parameters**	**Ex. time (hours)**	**CPU time (hours)**	**Memory peak (Gigabytes)**
SonicParanoid2	default	-t 128	0.83	70.06	61.79
SonicParanoid2	fast	-m fast -t 128	0.55	34.20	43.76
SonicParanoid2	sensitive	-m sensitive -t 128	4.28	496.42	156.09
SonicParanoid2	default (graph-only)	-go -t 128	0.66	58.57	53.04
SonicParanoid2	fast (graph-only)	-m fast -go -t 128	0.37	22.26	45.06
SonicParanoid2	sensitive (graph-only)	-m sensitive -go -t 128	4.11	485.35	154.12
Broccoli	default	-threads 128	2.98	65.74	275.35
OrthoFinder	default	-t 128	1.37	66.81	36.85
OrthoFinder (MSA)	MSA	-M msa -t 128	9.94	397.97	127.44
ProteinOrtho	default	-p=diamond -cpus=128	0.70	26.07	13.40
**Input dataset: 200 eukaryotes**					
**Tool**	**Settings**	**Extra Parameters**	**Ex. time (hours)**	**CPU time (hours)**	**Memory peak (Gigabytes)**
SonicParanoid2	default	-t 128	5.74	611.32	NA
SonicParanoid2	fast	-m fast -t 128	3.07	271.17	NA
SonicParanoid2	sensitive	-m sensitive -t 128	38.85	4,829.26	NA
SonicParanoid2	default (graph-only)	-go -t 128	5.06	551.30	NA
SonicParanoid2	fast (graph-only)	-m fast -go -t 128	2.39	211.49	NA
SonicParanoid2	sensitive (graph-only)	-m sensitive -go -t 128	38.21	4,772.33	NA
Broccoli	Terminated since it used all the available system memory (2 Terabytes)				
OrthoFinder*	default	-t 128	9.49	574.71	NA
OrthoFinder (MSA)*	MSA	-M msa -t 128	39.75	1,920.36	NA
ProteinOrtho	default	-p=diamond -cpus=128	6.49	217.07	NA
**Input dataset: 2000 MAGs**					
**Tool**	**Settings**	**Extra Parameters**	**Ex. time (hours)**	**CPU time (hours)**	**Memory peak (Gigabytes)**
SonicParanoid2	default	-t 128	41.60	4,882.26	NA
SonicParanoid2	fast	-m fast -t 128	25.03	2,737.93	NA
SonicParanoid2	default (graph-only)	-go -t 128	35.96	4,070.69	NA
SonicParanoid2	fast (graph-only)	-m fast -go -t 128	22.04	2,428.74	NA
Broccoli	Terminated after 6 days in which it used the 2 terabytes of system memory				
OrthoFinder	ERROR: too many open files				
OrthoFinder (MSA)	ERROR: too many open files				
ProteinOrtho*	default	-p=diamond -cpus=128	136.82	3,112.42	NA

Table S3: Execution times, CPU-times and memory usage for the tested tools. For the four tools described in this study the execution times and CPU times were measures on the four test datasets. Memory usage peaks for processing the QfO dataset were also estimated. Runs in which the tool name bears the "*" symbol originally failed, and required special intervention from the user side (Supplementary Text).

Pair	Sequence count sp1	Sequence count sp2	Sequence count difference folds (assuming sp1<sp2)	Proteome size sp1	Proteome size sp2	Proteome size difference folds (assuming sp1<sp2)	Mean protein length A	Mean protein length B	Fastest
A-B	3,422	2,236	-1.53	769,203	739,153	-1.04	224.78	330.57	0
B-A	2,236	3,422	1.53	739,153	769,203	1.04	330.57	224.78	1
A-C	3,422	2,353	-1.45	769,203	637,663	-1.21	224.78	271.00	0
C-A	2,353	3,422	1.45	637,663	769,203	1.21	271.00	224.78	1
A-D	3,422	1,889	-1.81	769,203	537,042	-1.43	224.78	284.30	0
D-A	1,889	3,422	1.81	537,042	769,203	1.43	284.30	224.78	1

Table S4: AdaBoost training samples. The first column shows the pairs of proteomes each sample represents (not used in the training). Columns 2–9 show training features for AdaBoost, which include information extracted from the input proteomes, such as protein count, proteome size, and average protein length. Last column shows the training labels.

Tool	Version	Mode	MMseqs	Diamond	BLAST
SonicParanoid	2.0.0	Fast	-s 2.5	mid-sensitive	X
SonicParanoid	2.0.0	Default	-s 4.0	sensitive	default
SonicParanoid	2.0.0	Sensitive	-s 6.0	very-sensitive	X
SonicParanoid	2.0.0	Most-sensitive	-s 7.5	ultra-sensitive	X
Broccoli	1.2.1	Default	X	more-sensitive	X
OrthoFinder	2.5.4	Default	X	more-sensitive	X

Table S5: Mapping of sensitivities of alignment tools to sensitivity settings of the tested orthology inference tools. Cells filled with "X" represent unavailable or untested settings.

MMseqs2						
**Mode**	**Complete alignments**	**Essential alignments**	**AdaBoost accuracy (%)**	**Avg. (%) used in essential sets**	**Saved hours**	**Saved time (%)**
most-sensitive	6.76	3.91	95.94	33.45	2.85	42.18
sensitive	2.57	1.65	94.91	31.22	0.91	35.60
default	0.64	0.47	94.61	25.50	0.17	25.84
fast	0.33	0.26	92.77	20.22	0.07	20.87
**Diamond**						
**Mode**	**Complete alignments**	**Essential alignments**	**AdaBoost accuracy (%)**	**Avg. (%) used in essential sets**	**Saved hours**	**Saved time (%)**
most-sensitive	2.35	1.71	95.37	31.94	0.63	27.01
sensitive	0.62	0.45	95.20	31.42	0.17	27.69
default	0.46	0.30	96.10	29.71	0.16	34.29
fast	0.23	0.16	96.70	26.78	0.07	31.05
**BLAST**						
**Mode**	**Complete alignments**	**Essential alignments**	**AdaBoost accuracy (%)**	**Avg. (%) used in essential sets**	**Saved hours**	**Saved time (%)**
default	18.41	13.32	83.38	33.83	5.09	27.65

Table S6: Saved execution time using AdaBoost-enhanced graph-based algorithm when processing QfO 2020 dataset. Results for 18 trials of SonicParanoid2 using the graph-based pipeline are shown. Sub-tables show the results obtained using (from top to bottom) MMseqs2, Diamond, and BLAST at different sensitivities. Columns two and three show the time required to perform homology searches (in hours) using the conventional all-versus-all (complete alignments) and the novel approach (essential alignments), respectively. Accuracies for the AdaBoost classifier and the average sizes of the original input proteins used in the essential subsets are shown in columns four and five, respectively. The last two columns on the right show the amount of time saved using the novel graph-based approach (in hours and percentage, separately).

Alignment tool	SonicParanoid mode	AdaBoost model	Ex. time (hours)	Saved hours	Saved time (%)	Saved time decrease (%)
MMseqs2	most-sensitive	Correct	3.91	2.85	42.18	-70.34
		Wrong	5.91	0.85	12.51	
	sensitive	Correct	1.65	0.91	35.60	-58.68
		Wrong	2.19	0.38	14.71	
	default	Correct	0.47	0.17	25.84	-53.30
		Wrong	0.56	0.08	12.07	
	fast	Correct	0.26	0.07	20.87	-38.81
		Wrong	0.29	0.04	12.77	
Diamond	most-sensitive	Correct	1.71	0.63	27.01	-23.11
		Wrong	1.86	0.49	20.77	
	sensitive	Correct	0.45	0.17	27.69	-37.29
		Wrong	0.51	0.11	17.36	
	default	Correct	0.30	0.16	34.29	-47.37
		Wrong	0.38	0.08	18.05	
	fast	Correct	0.16	0.07	31.05	-52.65
		Wrong	0.19	0.03	14.70	
BLAST	default	Correct	13.32	5.09	27.65	-12.17
		Wrong	13.94	4.47	24.29	

Table S7: Importance of correctly predicting the fastest inter-proteome alignments. To evaluate the importance of AdaBoost, we performed trials using a "wrong" model, which outputs the opposite prediction of the “correct” one. For example, if the model has an accuracy of 95.94% using MMseqs in the most-sensitive mode, then the "wrong" model will have an accuracy of 4.06%. Column four shows the total execution times for the homology searches using the “correct” or “wrong” AdaBoost classifier. The rightmost column shows the decrease in percentage of saved time when using the wrong model.

SonicParanoid default; Diamond [very-sensitive] AdaBoost ex. time 0.45h			
**Metric**	**Ex. time (hours)**	**% slower than AdaBoost**	**folds slower than AdaBoost**
Mean	0.4916	7.0877	1.0709
Max	0.4926	7.3142	1.0731
Min	0.4901	6.7669	1.0677
**SonicParanoid fast; Diamond [mid-sensitive] AdaBoost ex. time 0.17h**			
**Metric**	**Ex. time (hours)**	**% slower than AdaBoost**	**folds slower than AdaBoost**
Mean	0.1857	10.6744	1.1067
Max	0.1870	11.4419	1.1144
Min	0.1847	10.0791	1.1008
**SonicParanoid sensitive; MMseqs [-s 7.5] AdaBoost ex. time 3.87h**			
**Metric**	**Ex. time (hours)**	**% slower than AdaBoost**	**folds slower than AdaBoost**
Mean	4.7629	22.7122	1.2271
Max	4.7904	23.4210	1.2342
Min	4.7432	22.2058	1.2221

Table S8: Execution for predicted fastest pairs VS random selection of inter-proteomes. 10 runs for each of the three sensitivity settings were performed in which the inter-proteome alignment to be performed first was selected randomly. The average, maximum, and minimum execution times obtained using random selection were always slower than runs in which the fastest pair was predicted using AdaBoost.

Alignment tool	Complete alignments	Essential alignments	AdaBoost accuracy (%)	Avg. (%) used in essential sets	Saved hours	Saved time (%)
MMseqs2	67.38	56.48	83.89	28.09	10.90	16.18
Diamond	31.65	23.74	91.37	42.96	7.91	24.99

Table S9: Saved execution time on 2,000-MAG dataset using the novel graph-based approach at default sensitivity settings. Columns two and three show the time required to perform the homology searches (in hours) using the conventional all-versus-all (complete alignments) approach and the novel approach (essential alignments), respectively. The AdaBoost accuracies and the average sizes of the original input proteins used in the essential subsets are listed in columns four and five, respectively. The last two columns on the right show the amount of time saved (in hours and percentage) when compared with the complete alignments.

Alignment tool	SonicParanoid mode	Ex. time (hours)	Speed-up folds on Broccoli	Speed-up folds on OrthoFinder (MSA)
**MMseqs**	most-sensitive	4.17	-1.51	2.44
	sensitive	1.91	1.45	5.33
	default	0.75	3.69	13.57
	fast	0.55	5.01	18.44
**Diamond**	most-sensitive	1.93	1.43	5.27
	sensitive	0.66	4.17	15.33
	default	0.52	5.32	19.57
	fast	0.38	7.31	26.90
**BLAST**	default	13.67	-4.94	-1.34

Table S10: Speedup folds on Broccoli and OrthoFinder (MSA) when processing QfO dataset. Speedups refer to trials performed on SonicParanoid2 using only the graph-based algorithm and include the creation of ortholog groups. OrthoFinder (MSA) and Broccoli used Diamond to perform the alignments, whereas SonicParanoid was executed using different alignment tools (Supplementary Table S3). Negative values represent settings for which SonicParanoid was slower.

	Classification method from QfO benchmarking service							
	Diagonal quartiles		K-means		Square quartiles		Aggregate rank	
**SP2 settings**	**Rank**	**Rank change**	**Rank**	**Rank change**	**Rank**	**Rank change**	**Rank**	**Rank change**
SP2 (fast)	9th	10	4th	14	25th	-9	9th	11
SP2 (g) (fast)	19th		18th		16th		20th	
SP2	3rd	7	2nd	10	23rd	-19	2nd	8
SP2 (g)	10th		12th		4th		10th	
SP2 (sens)	1st	3	1st	9	24th	-21	1st	3
SP2 (g) (sens)	4th		10th		3rd		4th	

Table S11: Improvements in the QfO ranking due to the inclusion of domain-based orthology. Labels containing “(g)” represent SonicParanoid2 runs in which only graph-based orthology was performed. The columns “Rank change” contain the change in number of positions resulting from the inclusion of the domain-aware algorithm. Cells with green and red backgrounds respectively represent gains and losses in the ranks.

Species	Common name	Ortholog (UniProt ID)	Architecture in SonicParanoid2	Cosine sim. with PF0A8F4	Cosine sim. with PF0A8F0
Branchiostoma floridae	Florida lancelet	C3ZI77	52 PF00485 32 PF14681	0.563	0.666
Ciona intestinalis	Sea squirt	F6U8B5	70 PF00485 44 PF14681	0.619	0.702
Danio rerio	Zebrafish	A0A2R8Q5J9	91 PF00485 40 PF14681 16	0.633	0.72
Danio rerio	Zebrafish	E9QGS0	93 PF00485 40 PF14681 15	0.664	0.694
Danio rerio	Zebrafish	B0S7A4	79 PF00485 40 PF14681 21	0.623	0.646
Dictyostelium discoideum	Slime mold	Q55EL3	72 PF00485 40 PF14681	0.637	0.722
Gallus gallus	Chicken	A0A1D5PCN9	90 PF00485 35 PF14681 14	0.668	0.686
Gallus gallus	Chicken	A0A1L1RJC9	99 PF00485 40 PF14681 15	0.657	0.686
Gorilla gorilla	Gorilla	G3QI51	100 PF00485 39 PF14681 16	0.689	0.701
Homo sapiens	Human	Q9NWZ5	100 PF00485 40 PF14681 16	0.655	0.713
Lepisosteus oculatus	Spotted gar	W5MBZ5	87 PF00485 40 PF14681 16	0.595	0.706
Monodelphis domestica	Gray opossum	A0A5F8GEJ6	103 PF00485 41 PF14681 15	0.629	0.641
Mus musculus	Mouse	Q91YL3	100 PF00485 40 PF14681 16	0.655	0.713
Oryzias latipes	Medaka	A0A3B3HHD8	78 PF00485 40 PF14681	0.662	0.679
Oryzias latipes	Medaka	A0A3B3HWV8	85 PF00485 41 PF14681 16	0.599	0.699
Oryzias latipes	Medaka	H2LXZ5	100 PF00485 43 PF14681 15	0.656	0.685
Pan troglodytes	Chimpanzee	K7CT77	100 PF00485 40 PF14681 16	0.655	0.713
Rattus norvegicus	Rat	D3ZYQ8	100 PF00485 40 PF14681 16	0.655	0.713

Table S12: Domain-orthologs of eukaryote uridin–ecytidine kinase proteins recovered using SonicParanoid2. Third column shows a list of proteins predicted as orthologous to the human uridine–cytidine kinase proteins (Q9NWZ5), which was shown as an example of domain-fusion event by Persson *et al.* 2019. Column four shows the architectures of each protein as they appear in the training corpus for the Doc2Vec model. The last two columns show the cosine similarities assigned by Doc2Vec to each eukaryote uridine–cytidine kinase ortholog for *E. coli* PF0A8F4 and PF0A8F0 proteins, respectively.
