# Supplementary material for: SonicParanoid2: fast, accurate, and comprehensive orthology inference with machine learning and language models
Source: Genome Biol. 2024 Jul 25;25:195. doi: 10.1186/s13059-024-03298-4 (PMC11270883; doi:10.1186/s13059-024-03298-4)
Supplement: Supplementary file 4 — Additional file 4. Peer review history. [file 13059_2024_3298_MOESM4_ESM.docx]

**Review history**

**First round of review**

**Reviewer 1**

Orthology inference is a long-standing problem for which several tools have been developed from two decades ago. This shows the importance of the problem and its broad applications. Thus, it is of great importance to improve the speed of tools and their accuracy. Cosentino and Iwasaki reported a major update to the SonicParanoid tool for inferring orthologous genes. The innovation is to use AdaBoost to efficiently avoid unnecessary pairwise alignment and Doc2Vec for recovering missed orthologs based on pfam protein domains.

The manuscript is well written overall and the authors performed a comprehensive benchmarking. I was able to install and run SonicParanoid easily. The webpage of software includes sufficient information on installation, input, and output. The code on GitLab is publicly available and the team actively supports users via the issue tracker.

However, I have some comments to clarify the manuscript and results:

Major comments
1- I was not able to reproduce the results in terms of run time. When I ran SonicParanoid2 and OrthoFinder, I got different timing for orthofinder (detailed at the end of comment). I couldn't see this statement "On the QfO dataset, SonicParanoid2 was 18X .. faster than OrthoFinder". The exact command line of each tool should be mentioned in the method section. Besides, wall-clock time, CPU hours, and the needed memory for each tool/analysis should be reported clearly.

2- It is needed to have a run time comparison between tools on a large scale Eukaryotic datasets. When I tried it, orthofidner was faster than SonicParanoid2.

3- To showcase the superiority of adaptive gradient boosting in predicting the faster alignment between A-B and B-A, it would be great to compare it with a random approach where A-B or B-A is chosen randomly.

4- Doc2Vec was trained on QFO and MAG datasets and was tested on the same dataset if I understand it correctly. I'm wondering how it works on a new dataset. Would it be possible to have separate test/training/validation datasets as it is common in ML literature.

5- It would be great to compare the run time of SonicParanoid2 with Proteinortho6.

Minor comments:
6- Being able to use the output of diamond/mmseqs from another analysis would be a great feature for sonicparanoid2. This makes it possible to use as many CPUs as possible in an HPC rather than being limited to one machine. However, there must have been some technical limitations that this was not implemented.

7- I'm wondering whether it is possible to resume the sonicparanoid2 for a failed run due to a power issue for example. Having checkpoints would be a great feature (not necessary to have).

8- Please provide the definition of Orthologous groups/genes in the manuscript.
9- In Figure 2a, I couldn't find the point for OrthoMCL. it seems that two subplots of 2a correspond to two different tests (generalized and smaller SDT) which could be mentioned in the caption.
10- All analyses of the manuscript should be reproducible, and their scripts should be available (e.g. code for three classification methods for ranking)
11- Regarding "OrthoFinder and Broccoli failed to complete the orthology inference on the MAG dataset within eight days", it would be better to mention until which step they proceed if you still have the log.
12- In Table S1, it is not clear enough that the column "setting" refers to the alignment tool. The caption or table header could be improved.
13- It would be better to describe in detail how the stats of Fig 2 d are calculated, specifically the three classification methods should be described in more detail.
14- In Figure S11, the output could be mentioned in the flowchart .
15- It would be better to mention what (g) means in captions.
16- About Figure 3, I'm wondering why all cases have AAI > 30?
17- In Figure 4 and S16, removing boxes around the protein names (first column) could improve the clarity. Now they look like domains.
18- It might be needed to have a discussion/justification in the manuscript on the lower precision of the proposed method in the SwissTree benchmark.
19- Figure S21 might need a bit more description, what are the l and m in this case, and why 6 rows (is it only an example?).
20- I was checking the output of pairwise orthologs and its format is not clear. The header has four items but other lines have eight columns. Since the folder name is pairwise, I would expect to see two protein names per line (other float/integer values are also ok to have but in a reasonable order):
head -n 2 my_run/pairwise_orthologs/MAIZE.fa/MAIZE.fa-PARTE.fa
Size Relations OrthoA OrthoB
3 2 tr|A0A1D6LLK8|A0A1D6LLK8_MAIZE 1 tr|K7W637|K7W637_MAIZE 0.972 tr|A0C766|A0C766_PARTE 1

21- It would be also interesting to have a summary of main part of Sonicparanoid algorithm algorithm (probably in supplementary). As in this manuscript Sonicparanoid2, we are referred to Sonicparanoid1 and in 2019 publication we are referred to the inparanoid publication.

-- My experience in capturing run time: I ran SonicParanoid (2.0.4) and orthofinder (2.5.4) on the QFO dataset of 78 species using the following command lines on a Slurm computation node with 48 CPUs and 450GB of memory.
sonicparanoid -i qfo -o output -p qfo -t 48
orthofinder -f qfo -t 48 -a 16

Orthofinder needed 44 CPU hours and wall-clock of 75mins using 47 GB of memory (reported by seff in ubuntu). On the other hand, sonicparanoid2 needed 45.5 CPU hours and wall-clock of 77 mins using 225GB of memory. Using --mode fast, sonicparanoid2 needed 40 mins (20 CPU hours) with 239 GB of memory.

Besides, for 200 uniprot eukartotes, Orthofinder needed 440 CPU hours but sonicparanoid2 needed 554 CPU hours.

**Reviewer 2**

Accurate orthology inference is crucial in the field of comparative genomics, and SonicParanoid has proven to be a significant and evolving tool in this domain. Its consistent updates have continually improved its performance, particularly demonstrated through the Quest for Orthologs (QfO) benchmark. The latest version, SonicParanoid2, marks a substantial advancement in both scalability and accuracy. This is achieved by optimizing the directionality of alignment computations (A-B versus B-A) and incorporating domain architecture into the inference of orthology.

The manuscript is well-written and clear, and I would be happy to see it published. However, I have several questions and suggestions regarding the methodology that I think would enhance the manuscript if addressed.

1)

The authors have stated that if A-B is predicted to be faster, SonicParanoid2 conducts the A-B alignment first and then creates two subsets, ess(A) and ess(B), which include only proteins with alignment scores above a specified threshold. Since fully aligning A-B is equivalent to aligning B-A, subsequently aligning ess(B) to ess(A) seems unnecessary. All the scores required to compute the BBH are already available. It is important to clarify whether maybe the method stores only the best hit from the A-B alignment, and not all? Without this information, the need for multiple alignments is perplexing. Given that, if the alignment is indeed all-to-all maybe temporary storing all the alignments, instead of all the highest one and processing them to get BBH might be faster than the redundant alignment of the B-A (even for the smaller "essential" subset).

2)

The authors have chosen a set of features to predict the faster alignment between proteomes, including protein count, mean protein length, proteome size (count*length), and ratios derived from these variables. However in practice the only independent variables are the 1) protein count and 2) the average protein length, with the other features being redundant or derived directly from them. Maybe that a simpler machine learning method could potentially be as effective, especially if it is sensitive to the relative ratios of these two primary features. The added complexity of an AdaBoost predictor may not be justified if the problem can be addressed by simpler heuristic approach. It would be good if the authors to explore and discuss whether such heuristic approach— choosing the alignment based on the proteome size or by protein count—could perform comparably to the AdaBoost method.

3)
The challenge of predicting domain-based orthology is well-recognized in the field, given its inherent complexity. SonicParanoid's attempt to address this issue is commendable, and the results presented in the manuscript are promising. However, the manuscript would be strengthened by a more comprehensive description of the training process, particularly concerning the QfO dataset's role in both training and subsequent QfO benchmarking phase. Specifically there is a need to clarify if the training and testing sets were segregated to ensure no domain overlap, which could lead to the method learning domain identities rather than generalizable features of orthology. This distinction is vital, especially outside the QfO benchmark, where a domain may convey different orthology information across various taxonomic ranges.

4)
Moreover, the authors have not provided a baseline for performance comparison. For instance, a simple heuristic that considers all proteins sharing at least one domain as orthologs could serve as an informative baseline. This would enable a clearer evaluation of the performance impact when domains are utilized naively versus the proposed sophisticated method.

5)
Additionally, while SonicParanoid appears to mitigate some issues related to domain reshuffling, the persistent challenge is that different domains can have distinct evolutionary trajectories. It's not merely about whether a protein falls or not into an orthology group, but rather how the protein's diverse domains may place it in multiple orthology groups from the evolutionary perspective of each of the domains. Domain-based orthology should mean treating domains as separate evolutionary entities with their own unique histories, not just as a means to merge (or split) a particular groups.

6)
The manuscript outlines several filtering criteria used to improve the computation time of the domain-based method:

1) The protein length of one architecture exceed 3X the length of the other.
2) Their protein coverages differ by more than 25%.
3) One of the two architectures has more than double the domains of the other.
4) The two architectures have no mutual domains.

While the intent to streamline computation is clear, the first three filters may inadvertently dismiss legitimate cases of domain reshuffling, particularly in events where different proteins have fused. Such fusion events can result in proteins whose lengths are more than three times that of their counterparts or which have substantially different domain counts. I would urge the authors to consider the potential exclusion of valid orthologous relationships due to these filters and to discuss the implications of these criteria on the sensitivity and specificity of the orthology predictions.

**Authors’ response to reviewers**

Referee: 1

Orthology inference is a long-standing problem for which several tools have been developed from two decades ago. This shows the importance of the problem and its broad applications. Thus, it is of great importance to improve the speed of tools and their accuracy. Cosentino and Iwasaki reported a major update to the SonicParanoid tool for inferring orthologous genes. The innovation is to use AdaBoost to efficiently avoid unnecessary pairwise alignment and Doc2Vec for recovering missed orthologs based on pfam protein domains.

The manuscript is well written overall and the authors performed a comprehensive benchmarking. I was able to install and run SonicParanoid easily. The webpage of software includes sufficient information on installation, input, and output. The code on GitLab is publicly available and the team actively supports users via the issue tracker.

However, I have some comments to clarify the manuscript and results:

1- I was not able to reproduce the results in terms of run time. When I ran SonicParanoid2 and OrthoFinder, I got different timing for orthofinder (detailed at the end of comment). I couldn't see this statement "On the QfO dataset, SonicParanoid2 was 18X .. faster than OrthoFinder". The exact command line of each tool should be mentioned in the method section. Besides, wall-clock time, CPU hours, and the needed memory for each tool/analysis should be reported clearly.

-- My experience in capturing run time: I ran SonicParanoid (2.0.4) and orthofinder (2.5.4) on the QFO dataset of 78 species using the following command lines on a Slurm computation node with 48 CPUs and 450GB of memory.

sonicparanoid -i qfo -o output -p qfo -t 48

orthofinder -f qfo -t 48 -a 16

Orthofinder needed 44 CPU hours and wall-clock of 75mins using 47 GB of memory (reported by seff in ubuntu). On the other hand, sonicparanoid2 needed 45.5 CPU hours and wall-clock of 77 mins using 225GB of memory. Using --mode fast, sonicparanoid2 needed 40 mins (20 CPU hours) with 239 GB of memory.

Besides, for 200 uniprot eukartotes, Orthofinder needed 440 CPU hours but sonicparanoid2 needed 554 CPU hours.

**Response (R1C1-major):**

First of all, we want to thank the referee for taking the time to review our manuscript. The First major comment contains multiple important issues, and we will try to answer them one-by-one.
We also thank the referee for providing the details of the tool versions and the parameters used, which greatly clarified the issue.

The results originally shown for OrthoFinder were obtained from running with the “-M msa” parameter (which was the setting reported in the public QfO benchmark). This was mentioned in the material and methods, but it was not clear from the tables. So, we have re-done the runs for OrthoFinder with and without the “-M msa” parameter and updated the tables accordingly. Supplementary Table S3 includes the following information for the main runs on the 3 datasets we use in the revised version of this manuscript:
- cpu-time
- wall-time
- memory usage (only for the QfO dataset)
- non-default parameters for each tool and run

CPU time and wall time for all experiments were measured with *hyperfine* <https://github.com/sharkdp/hyperfine>. The log files, execution times (in JSON from hyperfine), scripts, figures, and tables generated are publicly available at <https://gitlab.com/salvo981/sonic-manuscript>. The materials produced during this revision are placed in the directory *genome_biology_review_files/round1*. To estimate the memory usage, we used *memory_profiler* (https://pypi.org/project/memory-profiler). As the memory usage monitoring is very resource demanding, we only performed it for the QfO dataset. An explanation of the memory usage measurement was added to the supplementary text.

Regarding the execution times that the referee observed, we found that they may be partly due to the slightly older version of Diamond used by OrthoFinder. OrthoFinder v2.5.4 uses Diamond 2.0.5 while we used Diamond 2.0.12 throughout (it should be noted that the latest version of OrthoFinder has switched to Diamond 2.0.13). By changing just the Diamond version, we observed a ~10% change in OrthoFinder’s speed on 48 CPUs as follows:

| **Tool** | **Diamond Version** | **Wall Time (min)** | **CPU Time (hr)** |
| --- | --- | --- | --- |
| SonicParanoid2 (default) | 2.0.12 | 83.4 | 54.20 |
| OrthoFinder (default) | 2.0.12 | 117 | 56.67 |
| OrthoFinder (default) | 2.0.5 | 106 | 50.73 |

These results are close to those observed by the reviewer, although we still find SonicParanoid2 to be faster than OrthoFinder with Diamond 2.0.12 (based on the wall-time). Maybe there was a major algorithmic changes between the two versions of Diamond.
More details can be found in the aforementioned GitLab repository in the file
genome_biology_review_files/round1/tables/execution_time_comparison.ods [sheet name: sp2 vs of2 (qfo20) 48cpus]

We would like to clarify that we included CPU time in the supplementary but not in the main manuscript for several reasons. Firstly, because CPU time is not a good proxy for the total execution time, unless every tool uses a single CPU. In fact, our results showed that SonicParanoid2 completed the execution earlier than other tools, even though those exhibit much lower CPU times (e.g., ProteinOrtho). This happened because while many tools utilize only a subset of the requested CPU cores during some analysis stages, such as the end of the all-vs-all alignments, SonicParanoid2 is optimized to utilize all CPU cores during this step. Secondly, because all tools (Broccoli, OrthoFinder, and now ProteinOrtho) reported their execution times using the wall time.

2- It is needed to have a run time comparison between tools on a large scale Eukaryotic datasets. When I tried it, orthofidner was faster than SonicParanoid2.

**Response (R1C2-major):** We have compared all tools on a dataset of 200 eukaryotes (Figure S4, Table 3 and Supplementary Table S3). It should be noted that OrthoFinder initially failed to process the 200-eukaryote dataset after showing an error, “too many files being opened at the same time”. This issue is described in <https://github.com/davidemms/OrthoFinder/issues/571>. Hence, we manually increased the maximum number of files that can be opened at the same time in our servers. Unfortunately, we were not able to apply the same fix when testing OrthoFinder on the dataset with 2,000 MAGs, since our system administrators did not allow such change.

3- To showcase the superiority of adaptive gradient boosting in predicting the faster alignment between A-B and B-A, it would be great to compare it with a random approach where A-B or B-A is chosen randomly.

**Response (R1C3-major):** We performed a total of 33 runs using the 3 main settings of SonicParanoid2. For each setting, 10 random runs and a run using AdaBoost prediction were performed. As shown in Supplementary Table S8, AdaBoost prediction improves the speed by 7-22% depending on the sensitivity setting.

4- Doc2Vec was trained on QFO and MAG datasets and was tested on the same dataset if I understand it correctly. I'm wondering how it works on a new dataset. Would it be possible to have separate test/training/validation datasets as it is common in ML literature.

**Response (R1C4-major):**

The Doc2Vec model was used to generate the embedding for an architecture of a protein where individual annotated domains and unannotated regions are considered as words and their arrangement on the protein is considered a document. The model was trained with the bag of word strategy where the context (i.e., the surrounding words) are used to predict the central word. Then, only the encoder part of the model was used to produce the embedding for an input domain architecture.

It is important to note that this is an unsupervised approach that does not directly incorporate ortholog relationship of the domain into the embedding. There was no labeling for the training data. The Doc2Vec model is always freshly trained and then used on the same dataset to ensure that the model has seen all domains in the dataset (i.e., there is no unknown input). This design also enables the domain-based pipeline to adapt to any input proteome and domain annotation. We found that the Doc2Vec model can be trained very quickly (Supplementary Figure S2) and have little impact on the total computational cost, especially on large datasets (Table 2 and 3).

We have modified the main text to clarify these points.

**LINES: 229-234; 447; 462-467**

5- It would be great to compare the run time of SonicParanoid2 with Proteinortho6.

**Response (R1C5-major):** ProteinOrtho6 was published on December 13th 2023 while this manuscript was still under review. Also, to the best of our knowledge, there was no preprint available. So, it was not possible for us to include it. Nevertheless, we found that SonicParanoid2 is faster than ProteinOrtho6 especially on large datasets (Table 2 and 3), even though ProteinOrtho6 exhibits almost half the CPU time (as it performed only one inter-proteome alignment for each proteome pair). To our understanding, ProteinOrho6 writes a lot of small files, whose number exponentially grows with the size of the input dataset. This might explain the longer execution times observed on larger datasets, as well as the relatively higher proportion of system times (please check genome_biology_review_files/round1/tables/execution_time_comparison.ods) compared to other tools. ProteinOrtho6 used more than 1 TB of storage for the 2,000 MAGs analysis, while SonicParanoid2 (default mode) used only ~350 GB.

6- Being able to use the output of diamond/mmseqs from another analysis would be a great feature for sonicparanoid2. This makes it possible to use as many CPUs as possible in an HPC rather than being limited to one machine. However, there must have been some technical limitations that this was not implemented.

**Response (R1C6-minor):** We agree that it could be useful and might be implemented in the future. At present, SonicParanoid2 expects the tab-separated output of diamond/mmseqs/blast to have only 6 columns. So, we can modify SonicParanoid2 to be able to parse the default tab-separated output other formats. In fact, there was already an open issue, <https://gitlab.com/salvo981/sonicparanoid2/-/issues/51>. To minimize unintended problems during the review process, we have decided to freeze the SonicParanoid2 version. We hope the referee understands that we cannot implement this feature by this review round. Please follow the GitLab issue and the development branch, https://gitlab.com/salvo981/sonicparanoid2/-/tree/dev-release-206?ref_type=heads, for future feature updates.

7- I'm wondering whether it is possible to resume the sonicparanoid2

for a failed run due to a power issue for example. Having checkpoints

would be a great feature (not necessary to have).

**Response (R1C7-minor):** Yes. At each “update-run”, SonicParanoid2 checks if the required alignments have already been performed and will reuse them. To resume a run, the user can provide the same input files and specify the same output directory as a previous run. Then, by adding the parameter "-ot" (or --overwrite-tables), SonicParanoid2 would overwrite the species-species ortholog tables. For more details on the parameters, please run “sonicparanoid --help” or refer to the wiki on GitLab, [https://gitlab.com/salvo981/sonicparanoid2/-/wikis/h](https://gitlab.com/salvo981/sonicparanoid2/-/wikis/SonicParanoid)ome.

8- Please provide the definition of Orthologous groups/genes in the manuscript.

**Response (R1C8-minor):**

The general definition of orthology was provided at the start of the introduction together with references (**LINES: 39-40**). We have modified the results section to clarify the meaning of orthologous groups, as well as species-species ortholog clusters. **LINES: 81-85**

9- In Figure 2a, I couldn't find the point for OrthoMCL. it seems that two subplots of 2a correspond to two different tests (generalized and smaller SDT) which could be mentioned in the caption.

**Response (R1C9-minor):** In the GSTD LUCA panel, OrthoMCL is located at the coordinate (x: ~2500 y: ~0.4079) in the upper right corner. In the STD Bacteria panel, it is partially covered by the marker of SonicParanoid2 (fast) at coordinate (x: ~1600 y: ~0.66) in the upper right corner (notice the orange crosshair). We have modified the caption as suggested.

10- All analyses of the manuscript should be reproducible, and their scripts should be available (e.g. code for three classification methods for ranking)

**Response (R1C10-minor):** All the code are available through GitLab repository:

<https://gitlab.com/salvo981/sonic-manuscript>. Relevant scripts, logs and media files, generated for this revision were placed at the directory /sonic-manuscript/genome_biology_review_files/round1.

Regarding the code for the three classification methods, we have not developed the scripts. The public resource from the QfO benchmark web-page was used
(<https://orthology.benchmarkservice.org/proxy/results/2020/#tab_generalized-species-tree-discordance-benchmark_fungi_OEBD00200002AN>, just click on “Summary table” after you open the link).

We have reached out to Adrian Altenhoff at ETH Zurich, who is the creator and main developer of the QfO Benchmark, and he kindly provided us with the links to the relevant source codes.

The classification methods were developed as part of OpenEBench pipeline, and the relative code is available at <https://github.com/inab/OpenEBench_scientific_visualizer>. This is the same code used in the QfO benchmark page, <http://orthology.benchmarkservice.org/>.

The script to perform the clustering is available at
<https://github.com/inab/OpenEBench_scientific_visualizer/blob/master/src/classification.js>. The quartiles are generate using the d3’s square quartile method, while the clustering uses the “cluster” package from <https://www.npmjs.com/package/clusters>.

We have included this information also in the supplementary text.

11- Regarding "OrthoFinder and Broccoli failed to complete the orthology inference on the MAG dataset within eight days", it would be better to mention until which step they proceed if you still have the log.

**Response (R1C11-minor):**

Regarding Broccoli, when processing the 2,000 MAGs dataset, it got stuck at “STEP 3: network analysis”. Specifically, it used up all the 2TB of memory and caused the system to memory swap. It was ultimately killed after about 6 days. The same thing happened when Broccoli was tested on the dataset with 200 eukaryotes. We set a 2-days limit, but by then it had already used up all the 2TB of memory and caused the server to memory swap. It was terminated on the third day.

Regarding OrthoFinder, the log file indicates that it failed after the all-vs-all alignment step, which lasted about 6-7 days. It crashed with the same error mentioned in R1C2 (<https://github.com/davidemms/OrthoFinder/issues/571>). OrthoFinder v2.5.4 opens N^2 files when given N input proteomes, which would mean opening 4 million files in the case of the 2,000 MAGs dataset. In the case of the 200 eukaryotes dataset, which requires 40,000 alignment, we were able to increase the soft limit for file opening from 1,024 to 1,048,576.

It should be noted that for this revision, we downloaded OrthoFinder2 (v2.5.4) again (which is several commits more recent than the one we used earlier) and that this version of OrthoFinder2 produced the error message immediately after processing the input, instead of waiting until after completing the all-vs-all alignments. The newer version of OrthoFinder2 checks the file opening limit of the operating system in advance. We have updated the commit hash for OrthoFinder2 in the Materials and Methods accordingly.

ProteinOrtho6 initially failed on the 2,000 MAGs dataset because it does not allow the stop symbol “*” in the FASTA sequence. This is easily fixed by preprocessing the FASTA file, and we have notified the developer, <https://gitlab.com/paulklemm_PHD/proteinortho/-/issues/91>. We processed the input files to remove all “*” symbols in the sequences and reran ProteinOrho6. This time it completed the execution successfully after about 5.5 days (Table 2).

The issues discussed above, are also described in a paragraph in the Supplementary Text.

12- In Table S1, it is not clear enough that the column "setting" refers to the alignment tool. The caption or table header could be improved.

**Response (R1C12-minor):** We have revised the column name to Alignment Tool as suggested.

13- It would be better to describe in detail how the stats of Fig 2 d are calculated, specifically the three classification methods should be described in more detail.

**Response (R1C13-minor):** We have expanded the description of the three classification methods in the paragraph “Test datasets, accuracy benchmark and ranking“. **LINES: 337-344**. Additionally, the links to all the source files necessary reproduce our rankings are described in supplementary text.

14- In Figure S11, the output could be mentioned in the flowchart .

**Response (R1C14-minor):** We have modified the figure as suggested.

15- It would be better to mention what (g) means in captions.

**Response (R1C15-minor):** We have updated the captions of the 3 figures in which (g) appears.

16- About Figure 3, I'm wondering why all cases have AAI > 30?

**Response (R1C16-minor):** 30% is the default similarity threshold used to estimate the average amino acid similarity (AAI) for *comparem* (<https://github.com/donovan-h-parks/CompareM>). The command used was "comparem aai_wf --proteins --file_ext faa -c 128 --sensitive input/ output/".

We also tried reducing the threshold to 20% on a pair of distant proteomes (*C. elegans* vs *T. kodakarensis*) and observed a negligible change in AAI (38.99-39.95%). Based on this result, we do not expect any major difference due to the AAI threshold.

17- In Figure 4 and S16, removing boxes around the protein names (first column) could improve the clarity. Now they look like domains.

**Response (R1C17-minor):** We have modified the 2 figures as suggested.

18- It might be needed to have a discussion/justification in the manuscript on the lower precision of the proposed method in the SwissTree benchmark.

**Response (R1C18-minor):** We have updated the results section as suggested. **LINES: 236-245**

19- Figure S21 might need a bit more description, what are the l and m in this case, and why 6 rows (is it only an example?).

**Response (R1C19-minor):** We are sorry for the confusion. The caption has been revised. Regarding *l* and *m*, they represent the number of architectures (i.e., proteins with annotated domain architectures) in the two proteomes, A and B, respectively. So in the particular example in Supplementary Figure S21, *l* = 5 and *m* = 6.

20- I was checking the output of pairwise orthologs and its format is not clear. The header has four items but other lines have eight columns. Since the folder name is pairwise, I would expect to see two protein names per line (other float/integer values are also ok to have but in a reasonable order):

head -n 2 my_run/pairwise_orthologs/MAIZE.fa/MAIZE.fa-PARTE.fa

Size Relations OrthoA OrthoB

3 2 tr|A0A1D6LLK8|A0A1D6LLK8_MAIZE 1 tr|K7W637|K7W637_MAIZE 0.972 tr|A0C766|A0C766_PARTE 1

**Response (R1C20-minor):** We agree that the folder name is misleading. Each line of these files contains a cluster of orthologs and potential paralogs from the two species. In the above example, this ortholog groups consists of three total genes (the Size column), two genes from MAIZE, with relative scores 1.000 and 0.972 (the OrthoA column), and one gene from PARTE (the OrthoB column). This folder might be renamed “species-species-orthologs”. We will update SonicParanoid2 to ensure that the outputs are clear.
Following is the issue:
https://gitlab.com/salvo981/sonicparanoid2/-/issues/67

On a side note, the pairwise orthologs can be obtained by supplying the “--output-pairs” parameter to the program (in a new run or a previous run).

21- It would be also interesting to have a summary of main part of Sonicparanoid algorithm algorithm (probably in supplementary). As in this manuscript Sonicparanoid2, we are referred to Sonicparanoid1 and in 2019 publication we are referred to the inparanoid publication.

**Response (R1C21-minor):** We have added a short description of SonicParanoid in the Supplementary Text and would like to direct the readers to the full supplementary explanation (about 26 pages) in our 2019 publication:

<https://oup.silverchair-cdn.com/oup/backfile/Content_public/Journal/bioinformatics/35/1/10.1093_bioinformatics_bty631/3/bioinformatics_35_1_149_s1.pdf?Expires=1714522188&Signature=FwuT~W0zcKAezVxV3hK6Skim1HBoVjRzZ4R7Ew3PxyS6WS-nQCy8O89-xGDPjKJwXr7LxjxMsqR99F4mT91e-fTitMP8myRNq57DnzJpXgcOsSJz298nBxLIYuqF1fFUabZuijSG2a-kWOafLK92X2SK1645zFT~R7v7nbpYeoQXdIvCFcR0j8M1bPUdfFyEYPG3c9vesk61GJCvpIFti5z5iMHje6mtzcN7z6DJzsrNfISX2hgTIj9cTmYVO-5CKhgqS0zVaFpVArsRwMGZ1Hq0DLOlAgrwrJ5FDjeuq6At58sKl8b3Fovg1ioPzXkU~0WVNqk0H4biv8UGCLNcaQ__&Key-Pair-Id=APKAIE5G5CRDK6RD3PGA>

**Response to referee 2**

Reviewer #2: Accurate orthology inference is crucial in the field of comparative genomics, and SonicParanoid has proven to be a significant and evolving tool in this domain. Its consistent updates have continually improved its performance, particularly demonstrated through the Quest for Orthologs (QfO) benchmark. The latest version, SonicParanoid2, marks a substantial advancement in both scalability and accuracy. This is achieved by optimizing the directionality of alignment computations (A-B versus B-A) and incorporating domain architecture into the inference of orthology.

The manuscript is well-written and clear, and I would be happy to see it published. However, I have several questions and suggestions regarding the methodology that I think would enhance the manuscript if addressed.

First of all, we want to thank the referee #2 for the encouraging comments, and for taking the time to review our manuscript. Following we address the issues noted by the referee point-by-point.

1)

The authors have stated that if A-B is predicted to be faster, SonicParanoid2 conducts the A-B alignment first and then creates two subsets, ess(A) and ess(B), which include only proteins with alignment scores above a specified threshold. Since fully aligning A-B is equivalent to aligning B-A, subsequently aligning ess(B) to ess(A) seems unnecessary. All the scores required to compute the BBH are already available. It is important to clarify whether maybe the method stores only the best hit from the A-B alignment, and not all? Without this information, the need for multiple alignments is perplexing. Given that, if the alignment is indeed all-to-all maybe temporary storing all the alignments, instead of all the highest one and processing them to get BBH might be faster than the redundant alignment of the B-A (even for the smaller "essential" subset).

**Response (R2C1):** Exactly, we store only the best hits from the first inter-proteomes alignments. We have modified the main text to clarify this detail (**LINES: 150-151; 382-383**). Regarding the final part of the comment that the B-A alignment may not need to be performed, ProteinOrho6, a tool we included in this revision, completely skips the second inter-proteome alignment (B-A) and instead uses a heuristic to estimate the bitscores based on the content of A-B. We find this approach to be very promising (as we briefly discuss in the Conclusions paragraph), and we might explore in the future the possibility to integrate a similar approach in SonicParanoid **(LINES: 289-291)**.

2)

The authors have chosen a set of features to predict the faster alignment between proteomes, including protein count, mean protein length, proteome size (count*length), and ratios derived from these variables. However in practice the only independent variables are the 1) protein count and 2) the average protein length, with the other features being redundant or derived directly from them. Maybe that a simpler machine learning method could potentially be as effective, especially if it is sensitive to the relative ratios of these two primary features. The added complexity of an AdaBoost predictor may not be justified if the problem can be addressed by simpler heuristic approach. It would be good if the authors to explore and discuss whether such heuristic approach— choosing the alignment based on the proteome size or by protein count—could perform comparably to the AdaBoost method.

**Response (R2C2):** Thank you very much for the suggestion. Using the suggested heuristic based on proteome size or protein count turns out to be a very viable option. We found that the heuristic provides as good performance as the AdaBoost model when MMseqs or Diamond was used. When BLASTP was used, using AdaBoost yielded ~3% speed improvement.

Regarding the features for predicting the faster alignment, we originally included extra features, such as average molecular weight, that convey different information than proteome size. But these features were later removed during the feature selection step (data not shown). It should be noted that as the features that the AdaBoost model currently used are already computed in SonicParanoid for other purposes, there is minimal impact on the execution time for enabling this prediction step.

In the current SonicParanoid version, we decided to keep the AdaBoost predictor because in some instances (6-8% of the time) the proteome size and protein count disagree with each other. Here are two examples of such pairs:

I. scapularis	5,815,358aa	20,473 proteins

P. graminis	6,184,957aa	15,688 proteins

with proteome size and protein count ratios of 1.064 and -1.305, and

C. neoformans	3,563,227 aa	6,604 proteins

R. baltica	2,290,149 aa	7,271 proteins

with proteome size and protein count ratios of -1.556 and 1.101

Furthermore, we would like to do further testing before employing the heuristic (for example, by using larger and more diverse proteome datasets), and we would like to train the AdaBoost models using data from multiple aligners and settings. In the current study, the AdaBoost model was trained using only MMseqs’s execution times at the highest sensitivity settings.

We have modified the “Conclusions” and the “Material and Methods” paragraphs to reflect the findings. (**LINES: 286-289; 423-429**)

3)

The challenge of predicting domain-based orthology is well-recognized in the field, given its inherent complexity. SonicParanoid's attempt to address this issue is commendable, and the results presented in the manuscript are promising. However, the manuscript would be strengthened by a more comprehensive description of the training process, particularly concerning the QfO dataset's role in both training and subsequent QfO benchmarking phase. Specifically there is a need to clarify if the training and testing sets were segregated to ensure no domain overlap, which could lead to the method learning domain identities rather than generalizable features of orthology. This distinction is vital, especially outside the QfO benchmark, where a domain may convey different orthology information across various taxonomic ranges.

**Response R2C3):** A similar comment was given by referee 1. We are sorry that we did not describe how the Doc2Vec model was trained and used clearly enough. We have updated the Materials and Methods section to address this issue.

The Doc2Vec model was used to generate the embedding for an architecture of a protein where individual annotated domains and unannotated regions are considered as words and their arrangement on the protein is considered a document. The model was trained with the bag of word strategy where the context (i.e., the surrounding words) are used to predict the central word. Then, only the encoder part of the model was used to produce the embedding for an input domain architecture.

It is important to note that this is an unsupervised approach that does not directly incorporate ortholog relationship of the domain into the embedding. There was no labeling for the training data. The Doc2Vec model is always freshly trained and then used on the same dataset to ensure that the model has seen all domains in the dataset (i.e., there is no unknown input).This means that, for example, the model used for the QfO dataset is trained after the extraction of the architectures (block 2 in Figure 1b), used in the identification of candidate orthologs (block 3-4 in Figure 2b) and never used again. Furthermore, we could not leave out any domains from the training because the Doc2Vec model was trained using architectures which consist of Pfam ID and the length of unannotated regions as words. Therefore, the model would not be able to generate an embedding for an unseen architecture.

We also would like to highlight that the documents (architectures) considered by the Doc2Vec model also include unannotated regions that could not be matched through a profile search. In the example depicted in Supplementary Figure S22c, the Document [78 PF00485 40 PF14681] contains “words” representing the unannotated regions in “78” and “40” as well as the two PFam domain IDs. Such information can be useful to for identifying similarity across architectures with unannotated regions at the same positions.

As mentioned in the response to comment R2C5 below, we appreciate the referee’s suggestions about how to better incorporate orthology information of the domains into our approach. In the future work, we will consider a more comprehensive strategy to handle domain orthology information.

The changes made to the main text relevant to this comment can be found in
**LINES: 229-234; 447; 462-467**

4)

Moreover, the authors have not provided a baseline for performance comparison. For instance, a simple heuristic that considers all proteins sharing at least one domain as orthologs could serve as an informative baseline. This would enable a clearer evaluation of the performance impact when domains are utilized naively versus the proposed sophisticated method.

**Response R2C4):** We performed a small comparison of the ortholog relations for the E.coli-H.sapiens pair to explore the impact of cosine similarity calculated from the embeddings and a baseline approach that counts the number of shared domain(s).

The histogram above shows the distribution of cosine similarity scores for pairs of architectures (candidate orthologs) that passed the prefiltering step (Figure 2b, block 3) and was used to populate the pairwise comparison matrix described in Supplementary Figure S23. The scores for pairs that share various numbers of domains were displayed. It should be noted that architectures that do not share any domain have low cosine similarity (<0.5) and were discarded by the algorithm. There is a positive correlation between the number of shared domain and cosine similarity as expected.

Here is an example of a cluster of ortholog relationships between E. coli and human that was obtained by merging the graph-based result with candidates from the domain-based algorithm. These protein pairs all share the same single domain (based on annotation through profile search, the right panel). In the left panel, “n/a” represents pairs of architectures that were rejected during the filtering step or because they have cosine similarities <0.5. Because the number of shared domain is the same, it is clear that cosine similarity allows for a higher resolution of comparison. This is because the cosine similarity score also accounts for the unannotated regions in the architectures. For example, some pairs of architectures may share the same domains but have different patterns of unannotated regions (different lengths and/or in different locations of the architectures). Such differences would lower the cosine similarity but not the number of shared domains. It should be noted that the cosine similarity is 1.0 only if all the components of the architectures, including the unannotated regions, are exactly the same and appear in the same order on the proteins.

Here is another example in which some pairs of proteins share two domains and some share one domain. The cosine similarity score not only is the lowest for the pair that shares one domain (0.66) but also can distinguish other pairs that share two domains (with values ranging from 0.70-0.84).

5)

Additionally, while SonicParanoid appears to mitigate some issues related to domain reshuffling, the persistent challenge is that different domains can have distinct evolutionary trajectories. It's not merely about whether a protein falls or not into an orthology group, but rather how the protein's diverse domains may place it in multiple orthology groups from the evolutionary perspective of each of the domains. Domain-based orthology should mean treating domains as separate evolutionary entities with their own unique histories, not just as a means to merge (or split) a particular groups.

**Response R2C5):** We agree with the referee that domains have separate evolutionary histories. Emma Persson at Sonnhammer’s group did what the referee suggests in Domainoid (Person et al. 2019) and the InParanoidDB9 database (Persson and Sonnhammer 2023). In this work, we are still focusing on the graph-based approach as the main source of orthologs detection, and the domain-based algorithm was included mainly to mitigate some limitations of the BBH. Specifically, our current Doc2Vec approach derives the embedding for the architectures based solely on context relationship between annotated domains and unannotated regions and does not directly utilize known orthologous relationship of the domains.

We are also working on a separate project that focuses more specifically on domains. The machine learning techniques we are exploring are too computationally demanding and not well-tested enough to be integrated into the current version of SonicParanoid2. The referee’s suggestion to consider each domain’s evolutionary history could be incorporated to improve the informativeness of the embedding produced by the Doc2Vec model (or any other encoder) for the purpose of ortholog prediction.

6)

The manuscript outlines several filtering criteria used to improve the computation time of the domain-based method:

1) The protein length of one architecture exceed 3X the length of the other.

2) Their protein coverages differ by more than 25%.

3) One of the two architectures has more than double the domains of the other.

4) The two architectures have no mutual domains.

While the intent to streamline computation is clear, the first three filters may inadvertently dismiss legitimate cases of domain reshuffling, particularly in events where different proteins have fused. Such fusion events can result in proteins whose lengths are more than three times that of their counterparts or which have substantially different domain counts. I would urge the authors to consider the potential exclusion of valid orthologous relationships due to these filters and to discuss the implications of these criteria on the sensitivity and specificity of the orthology predictions.

**Response R2C6):** Yes, we agree that these filters might reject legitimate orthologs with domain shuffling, fusion, or other complex rearrangements. As seen in Supplementary Figure S6, the highest improvement obtained from the domain-based algorithm was on the STD Bacteria dataset, which tend to contain smaller domain architectures than those in the Fungi and Eukaryota datasets. Hence, our approach works well mostly on orthologs with simple architectural evolution. And as the referee noted in other comments, there are many ways to utilize domain information that will improve our work.

For the current work, we want to underline that SonicParanoid2 uses the graph-based result as the backbone for identifying the ortholog groups, and that the domain-based algorithm primarily supplements it. Specifically, we adopted a higher threshold for accepting the orthologous relationships predicted solely by the domain-based algorithm and allowed a lower threshold when the prediction is concordant with the graph-based results. We have updated the Results section to highlight the limitations noted by the referee. **LINES: 246-257**

Also, we would like to note that the members of the QfO community are discussing the possibility to include new tests to evaluate orthology inference at the domain level. For example, in the 2022 challenge (for which results are publicly available), a new test was added to score the domain architectures of the predicted orthologs using the approach described in Dosch et al. (2023). Under this test, the inclusion of the domain-based predictions in SonicParanoid2 increased both the precision and recall compared to when only the graph-based algorithm was used.

**Second round of review**

**Reviewer 1**

The manuscript has been significantly improved. I appreciate all the efforts made by authors to address comments on clarification of the method and results. Also the authors have added new rigorous analysis and compared sonicparanoid2 with another tool, and improved the reproducibility of the work.

Just one last comment:
In the main text the speed improvement is now reported as "5x, 2.5x, and 1.3x" compared to other methods ("18x and 5x" was mentioned previously). It is needed to update the abstract accordingly. The authors could simply remove the phrase "up to 18X" from the abstract.

I do not need to see the revised abstract and I support the publication of the manuscript.

**Reviewer 2**

The authors have adequately addressed the reviewers' questions and revised the manuscript as needed.
